# Patient-Reported Symptom Burden and Temporal Trends During the First 18 Months After Gynecologic Cancer Diagnosis: A Population-Based Study

**DOI:** 10.3390/cancers18162628

**Published:** 2026-08-14

**Authors:** Lauren Philp, Genevieve Bouchard-Fortier, Lilian T. Gien, Sarah E. Ferguson, Jennifer Croke, Madeline Li, Rinku Sutradhar, Qing Li, Antoine Eskander

**Affiliations:** 1 Division of Gynecologic Oncology, Department of Obstetrics and Gynecology, University of Toronto, Toronto, ON M5G 1E2, Canada; 2 Princess Margaret Cancer Centre, University Health Network, Sinai Health System, Toronto, ON M5G 2M9, Canada; 3 Odette Cancer Centre, Sunnybrook Health Sciences Centre, Toronto, ON M4N 3M5, Canada; 4 Institute for Clinical Evaluative Sciences (IC/ES), Toronto, ON M4N 3M5, Canada; 5 Department of Radiation Oncology, University of Toronto, Toronto, ON M5T 1P5, Canada; 6 Department of Psychiatry, University of Toronto, Toronto, ON M5T 1R8, Canada; 7 Division of Otolaryngology, Department of Surgery, University of Toronto, Toronto, ON M5S 3H2, Canada

**Keywords:** patient-reported outcomes, gynecologic cancer, ovarian cancer, health-related quality of life

## Abstract

**Objectives**: Our objectives were to evaluate symptom prevalence, severity, and temporal trends ≤ 18 months from gynecologic cancer (Gyn-Ca) diagnosis using the ESAS-r questionnaire and to identify predictors of elevated symptom burden. **Methods**: We performed a retrospective population-based study of newly diagnosed Gyn-Ca patients in Ontario, Canada, from January 2007 to March 2022 using linked provincial healthcare databases. Patients with ≥1 ESAS-r completed ≤18 months from diagnosis were included. Diagnosis groups were created using WHO ICD-O3 codes. ESAS-r data were linked to provincial healthcare databases using unique coded identifiers. Primary outcome was a severe score (≥7) on any symptom by month (30-day block) from diagnosis, and secondary outcome was a moderate–severe score (≥4). Multivariable logistic regression identified predictors of elevated scores, with *p* < 0.05 being significant. **Results**: A total of 35,559 of 59,175 diagnosed patients (59.5%) completed ≥1 ESAS-r, with 207,000 unique assessments. Moderate–severe (≥4) or severe (≥7) scores were most common for tiredness (62.6%, 33.5%) and poor wellbeing (60.2%, 28.5%). All scores improved over time, with anxiety ≥ 4 dropping from 48.1% at diagnosis to 24.2% at 18 months. Patients with uterine cancer had the lowest incidence of elevated scores. Older age, lower rates of material deprivation and increased time from diagnosis were protective against elevated scores. Higher stage and comorbidity were predictive of elevated scores across all symptoms. Patients with vulvar/vaginal and cervix cancers had the highest odds of elevated scores. **Conclusions**: Patients with newly diagnosed Gyn-Ca report significant symptom burden in the first 18 months after diagnosis. Stage, diagnosis, and level of comorbidity were predictive of elevated scores, whereas older age, material resources and time from diagnosis were protective.

## Simple Summary

This population-based study examined symptom burden in 35,559 women with gynecologic cancers in Ontario using over 207,000 Edmonton Symptom Assessment Scale (ESAS-r) questionnaires completed during the first 18 months after diagnosis. The authors found that symptom burden was highest at diagnosis and during the first six months of treatment, with fatigue and poor wellbeing being the most common moderate-to-severe symptoms. Although symptoms generally improved over time, many patients continued to experience significant symptoms up to 18 months after diagnosis. Higher cancer stage, greater comorbidity, and diagnoses of cervical or vulvar/vaginal cancer were associated with worse symptom burden, while older age, greater material resources, and longer time from diagnosis were protective factors. These findings highlight the substantial and persistent physical and emotional impact of gynecologic cancers and support the routine use of patient-reported outcomes to identify patients who may benefit from additional supportive care interventions.

## 1. Introduction

Patient-centered care necessitates that health-related quality of life be a critical consideration in decision-making. Patient-reported outcomes (PROs) are a validated and standardized method to obtain unique insight into the patient experience [1]. The noted association between PROs and decreased morbidity and mortality has driven the widespread adoption of PROs in oncology trials [2,3]. However, the use of PROs to evaluate gynecologic cancer patients in standard clinical practice remains in its infancy [4]. To date, many PRO studies in gynecologic oncology have focused on patients at the end of life, a time of heavy symptom burden [5,6]. However, those earlier in their disease course can also experience poorly controlled symptoms [7], leading to suffering as well as treatment interruptions, delays, or discontinuation, which may worsen patient survival [8,9]. While this type of “real-world” data is limited, patients with gynecologic cancer completing PROs in a non-trial environment have reported lower quality of life than that reported in randomized trials, suggesting that patients may suffer more than anticipated during their cancer course [4].

The Edmonton Symptom Assessment Scale-revised (ESAS-r) is a reliable and validated PRO tool which screens for the presence and severity of nine common and important cancer-related symptoms [10,11]. Its use was adopted into routine cancer care at all regional cancer centers (RCCs) and many non-RCCs in Ontario, Canada, in 2007. Studies using this data have described elevated symptom burden at the time of cancer diagnosis and primary treatment in other solid tumors [12,13]. However, an assessment of symptom burden using ESAS-r during this time in a clinical population of gynecologic cancer patients has not been studied.

Thus, the primary objective of this study was to evaluate symptom prevalence, severity, and temporal trends in patients with gynecologic cancer in the first 18 months after diagnosis and to identify predictors of moderate-to-severe symptom burden.

## 2. Materials and Methods

### 2.1. Study Design and Population

This is a retrospective population-based observational study of patients with newly diagnosed gynecologic cancer in Ontario, Canada, between January 2007 and March 2022. The World Health Organization (WHO) International Classification of Disease for Oncology, 3rd edition, was used to define groups of cancers: ovary/fallopian tube, cervix, endometrium/uterus and vulva/vagina (Table S1). Defined cancers were then identified using the Ontario Cancer Registry (OCR), which registers all new provincial cancer cases, capturing up to 95% of incident diagnoses [14].

Patients were included if they were age ≥ 18 at diagnosis, were female sex, had a valid Ontario health insurance number (OHIP), and had ≥1 ESAS-r assessment recorded at any time during the first 18 months after cancer diagnosis. Patients who died within the study period were also included. Patients were excluded if they had a second malignancy diagnosed between 5 years prior to 18 months after their gynecologic cancer diagnosis to isolate the effect of their gynecologic cancer on ESAS scores. Patients were also excluded if they had a diagnosis of gestational trophoblastic neoplasia due to small sample size. A follow-up period of 18 months was chosen to ensure that the full treatment period was captured for all patients in which both neo-adjuvant and adjuvant treatment approaches were used.

### 2.2. Data Sources

ESAS-r data were obtained from the Cancer Care Ontario (CCO) Symptom Management Reporting Database (SMRD), which contains ESAS data from outpatient oncology visits from 2007 onwards. The ESAS-r was implemented province-wide as a routine standardized symptom screening program in 2007 and is currently ongoing. Patients are prompted to complete the evaluation at each outpatient visit to a cancer center. This dataset was then linked other provincial administrative healthcare databases housed at IC/ES, which contain health records for all OHIP-eligible patients. IC/ES (previously known as the Institute for Clinical and Evaluative Sciences) is an independent, non-profit research institute whose legal status under Ontario’s health information privacy law allows it to collect and analyze healthcare and demographic data, without consent, for health system evaluation and improvement. Datasets were linked using unique coded identifiers and analyzed at IC/ES, ensuring all data confidentiality and privacy policies were adhered to. Additional data were obtained from the following databases: the Ontario Cancer Registry (OCR), which captures cancer diagnoses and stage; the Registered Persons Database (RPDB), which contains demographic and death data; the Ontario Marginalization Index (ON-MARG), which measures indices of marginalization including economic, ethno-racial, age and social marginalization based on census data; the Immigration Refugees and Citizenship Canada Permanent Resident Database (CIC), which contains information on immigration status; the Canadian Institute for Health Information Discharge Abstract Database (CIHI-DAD), which records information regarding hospital admissions; the Ontario Health Insurance Plan claims database (OHIP), which contains all physician claims for OHIP-covered health services; and the National Ambulatory Care Reporting System (NACRS), which records visits to hospitals and ambulatory care settings, including the emergency department. The use of the data for this project is authorized under section 45 of Ontario’s Personal Health Information Protection Act (PHIPA) and does not require review by a Research Ethics Board.

### 2.3. Outcomes

Our primary outcome was a self-reported severe symptom score (≥7) on any ESAS-r symptom by month, as defined by 30-day blocks from diagnosis. This cutoff has previously been validated for identifying significant symptom burden [13,15]. The ESAS-r is a validated patient-reported outcome instrument that evaluates nine separate cancer-related symptoms (Figure S1), with each symptom scored using a numerical rating system from 0 to 10 where higher scores indicate worse symptoms or overall wellbeing. Scores can be further grouped to define symptom categories: no symptoms (0), mild symptoms (1–3), moderate symptoms (4–6) and severe symptoms (7–10) [5,13]. Of note, the ESAS-r captures information on symptom severity but does not provide information regarding factors that may have influenced these symptoms, such as treatment or supportive care interventions. Our secondary outcome was a self-reported moderate-to-severe symptom score (≥4) on any ESAS-r symptom by month to identify clinically significant but potentially non-severe symptoms [13,15,16]. If an ESAS-r score ≥1 was recorded by the patient within a 30-day block, the highest score available was used for analysis [13], and only one ESAS-r assessment per patient per day was included. If a patient did not complete an ESAS-r assessment during a specific 30-day block, then scores for that month were not inputted for that patient.

### 2.4. Covariates

Covariates were chosen a priori based on their clinical relevance and potential to impact ESAS-r scores and were measured at diagnosis date. Age was defined categorically as follows: 18–39, 40–49, 50–59, 60–69, 70–79 and 80+. Cancer stage was defined categorically from 1 to 4 using the TMN AJCC staging system. Patients with ≥1 gynecologic cancer diagnosed on the same day were reviewed independently by the authors; a primary diagnosis was assigned based on known patterns of disease spread, and a stage was assigned based on the primary tumor. Socioeconomic status was defined using the material resources quintile, a measure of material deprivation scored in quintiles from 1 to 5, with 5 representing the highest level of material deprivation [17,18]. Immigration status was categorized into two groups based on the presence or absence of an immigration record. Comorbidity was categorized into five groups defined by the sum of the Johns Hopkins ACG system© Aggregated Diagnosis Groups (ADGs) at diagnosis using the Johns Hopkins ACG system©. These groups defined increasing levels of comorbidity from lowest (0–4) to highest (20+), with ≥10 considered to be a clinically significant level of comorbidity [19,20]. A five-year lookback of multiple databases (OHIP/DAD/SDS/NACRS-ED) was used to calculate the comorbidity score [19]. The ADG score was then categorized into a 5-level variable and was adjusted as a risk factor. The impact of diagnosis date was analyzed in two ways: by year of diagnosis from 2007 to 2022 and by time from diagnosis in 6-month increments.

### 2.5. Statistical Analysis

Descriptive statistics were used to analyze baseline characteristics. Patients who completed ≥1 ESAS-r assessment during the study period were compared to those who did not using univariable analysis to determine the generalizability of our results. Further analysis was then performed only on those patients with available ESAS-r assessments, including the evaluation of baseline characteristics by diagnosis group. Among these patients, we then described the proportion reporting a moderate (≥4) or severe (≥7) score on any component of the ESAS-r by 30-day block from diagnosis, and the results were stratified by diagnosis group. The results were then depicted graphically to analyze the trajectory of symptoms by time from diagnosis. Predictors of moderate-to-severe ESAS-r scores were investigated via multivariable logistic regression using the primary and secondary score outcomes at each month in which a score was reported to determine the odds (+95% confidence interval) of an elevated symptom score. Logistic regression using a generalized estimating equation approach with an exchangeable correlation structure was used to account for hierarchical clustering. The characteristics included in our model were age, TNM stage, diagnosis, material resources quintile, immigration status, comorbidity, and time from diagnosis. A *p*-value of <0.05 was considered significant.

## 3. Results

Between 2007 and 2022, 59,715 patients were diagnosed with a gynecologic malignancy in Ontario, with 35,559 patients (59.5%) completing ≥1 ESAS-r questionnaire. Of these, 30,274 completed 18 months of follow-up (85.1%), and the majority of patients had endometrial cancer (52.5%), while vulvar/vaginal cancers were the least represented (6.2%). The mean age at diagnosis was 61.2 ± 13.66 years, with 29.6% of patients diagnosed between 60 and 69. Additional characteristics are shown in Table 1. Patients with cervical cancer were significantly younger than the cohort mean (mean age of 49.9 ± 14.69 years), with 54.9% diagnosed between 18 and 49 (Table S2). Patients with vulvar/vaginal and ovarian cancers died at the highest rate during the study period, with 23% and 21.5% of patients dying, respectively (*p* < 0.001) (Table S2). Patients that did not complete an ESAS-r tended to be older, have endometrial cancer, and have higher marginalization and elevated comorbidity (Table S3). They were also more likely to die during the study period (Table S3). Overall, patients were less likely to complete an ESAS-r assessment at the beginning of the study period; however, completion rates increased yearly between 2007 and 2019. Completion rates then dropped significantly between 2020 and 2022, likely due to the COVID-19 pandemic (Table S3).

A total of 207,000 unique ESAS-r assessments were completed during the study period, with the highest completion rates in months 3 and 4 (39% and 38.9%, respectively) (Table S4). Overall, patients averaged 5.82 ± 5.99 monthly ESAS-r assessments over a mean of 4.19 ± 3.23 months, with ovarian cancer patients completing assessments at the highest rate (mean 7.38 ± 7.60 monthly assessments over mean of 5.35 ± 3.77 months), with 64.7% of patients completing ≥ 1 assessment.

Patients were most likely to report elevated scores for tiredness (≥4: 62.6% and ≥7: 33.5%) and poor wellbeing (≥4: 60.2%, ≥7: 28.5%) (Table 2A,B). Nausea was the least problematic symptom, with only 9.3% reporting a severe score at any time. Patients with ovarian, vulvar/vaginal and cervical cancers had a higher incidence of elevated scores as compared to patients with endometrium/uterine cancer (Table 2A,B).

**Figure 1:**
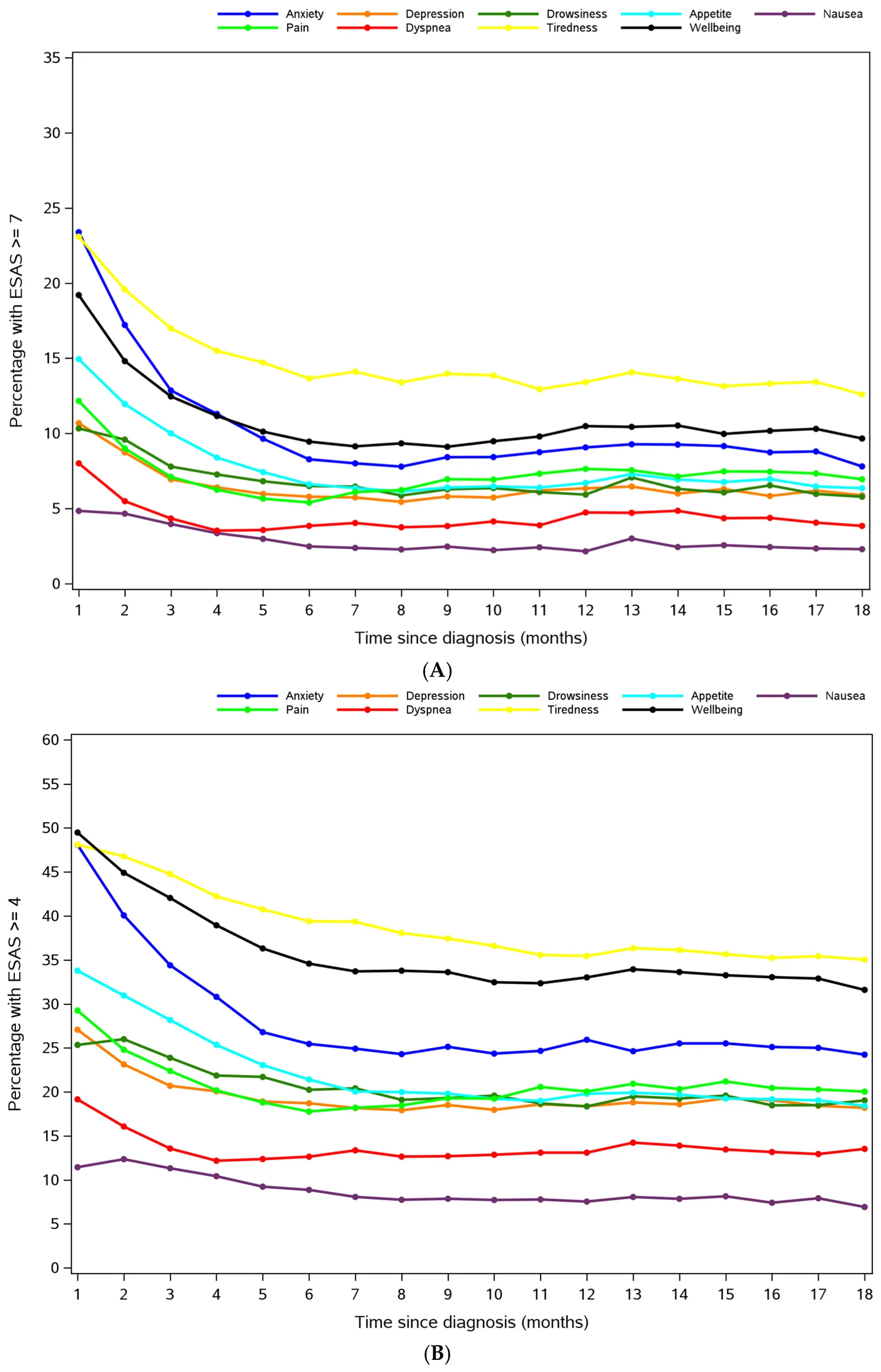
(**A**) Percentage of patients reporting severe scores (ESAS ≥ 7) by month from diagnosis (*n* = 35,559). (**B**) Percentage of patients reporting moderate-to-severe scores (ESAS ≥ 4) by month from diagnosis (*n* = 35,559).

**Figure 2:**
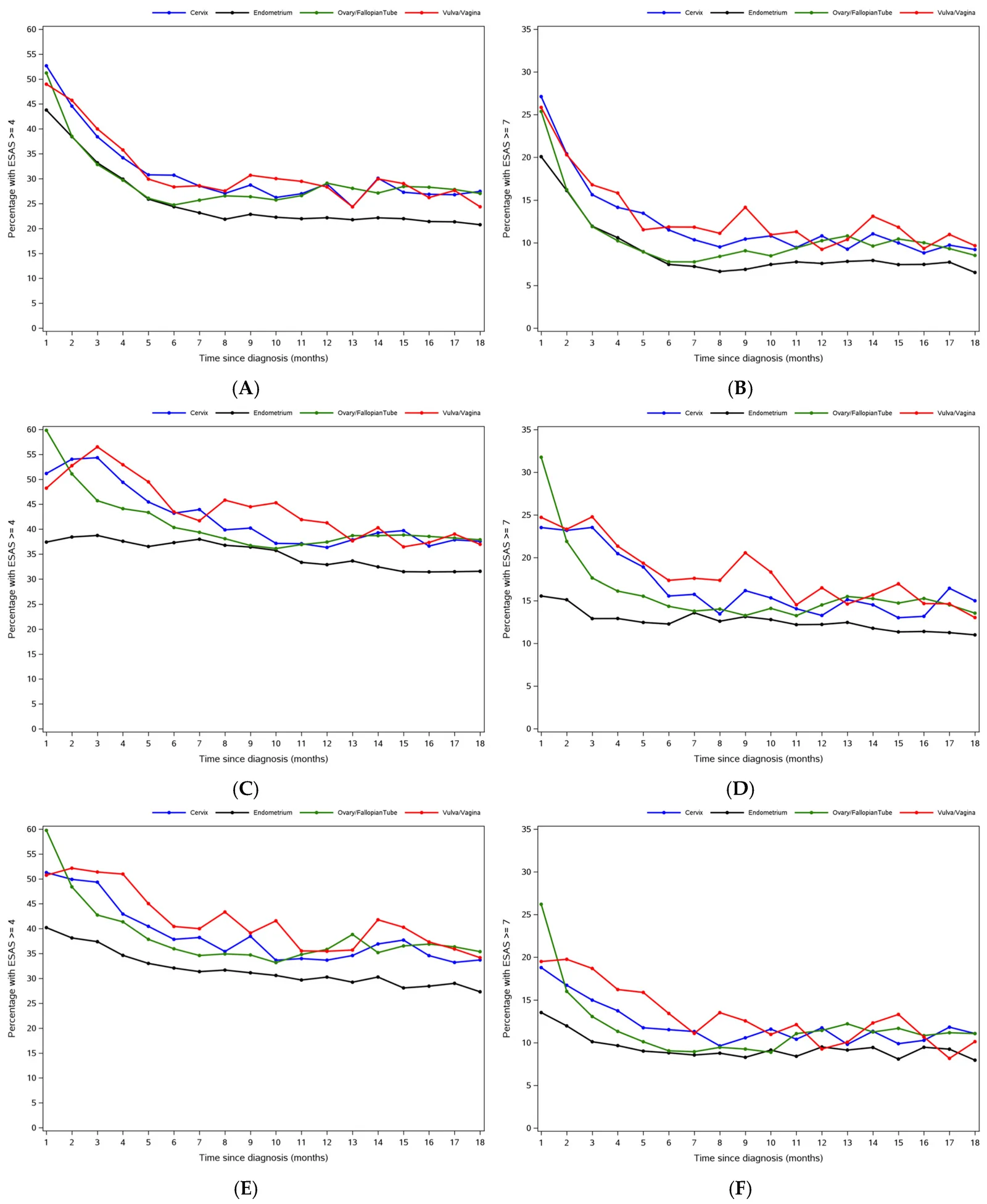

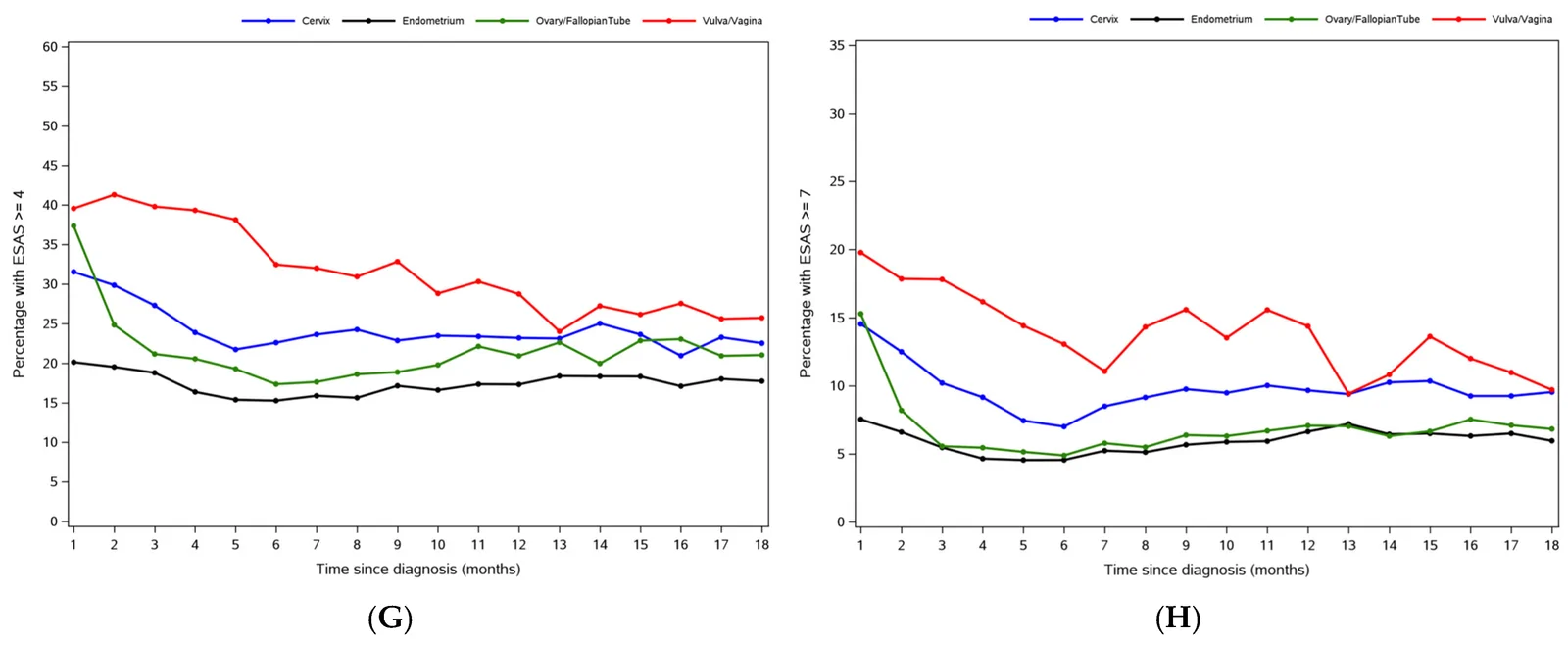
(**A**–**H**) Percentage of patients reporting moderate-to-severe (≥4) or severe scores (≥7) on ESAS symptoms by month from diagnosis (*n* = 35,559). (**A**) Percentage of patients reporting moderate-to-severe anxiety scores (ESAS ≥ 4) by month from diagnosis (*n* = 35,559); (**B**) percentage of patients reporting severe anxiety scores (ESAS ≥ 7) by month from diagnosis (*n* = 35,559); (**C**) percentage of patients reporting moderate-to-severe tiredness scores (ESAS ≥ 4) by month from diagnosis (*n* = 35,559); (**D**) percentage of patients reporting severe tiredness scores (ESAS ≥ 7) by month from diagnosis (*n* = 35,559); (**E**) percentage of patients reporting moderate-to-severe wellbeing scores (ESAS ≥ 4) by month from diagnosis (*n* = 35,559); (**F**) percentage of patients reporting severe wellbeing scores (ESAS ≥ 7) by month from diagnosis (*n* = 35,559); (**G**) percentage of patients reporting moderate-to-severe pain scores (ESAS ≥ 4) by month from diagnosis (*n* = 35,559); (**H**) percentage of patients reporting severe pain scores (ESAS ≥ 7) by month from diagnosis (*n* = 35,559).

Tiredness, wellbeing and anxiety were the symptoms with the highest incidence of moderate-to-severe scores by month (Figure 1A,B). Scores on all symptoms reduced with time from diagnosis, with the largest reductions noted in the first 6 months (Figure 1A,B). Most notably, anxiety scores ≥ 4 dropped from 48.1% at diagnosis to 24.2% at eighteen months. A similar trend was noted with anxiety scores of ≥7 (Figure 2A,B), whereas tiredness and poor wellbeing continued to be reported at high rates across the study period (Figure 2C–F). When each diagnosis group was evaluated separately, rates of moderate-to-severe and severe scores tended to be highest in the initial months after diagnosis and to drop for all symptoms across the study period (Figure S2A–H). Notably, vulvar/vaginal cancer patients had significantly higher pain scores than patients with other diagnoses (Figure 2G,H).

The odds of reporting an elevated ESAS-r score varied by patient and disease characteristics (Table 3A,B). Older age was protective against many moderate-to-severe symptoms, with older patients reporting lower levels of anxiety (OR 0.88), depression (OR 0.91), nausea (OR 0.88), pain (OR 0.97) and poor wellbeing (OR 0.97) (Table 3B). A similar trend was noted for severe scores (≥7) (Table 3A). Patients with higher cancer stage reported higher odds of both moderate-to-severe and severe scores for all symptoms, with the odds increasing as stage increased from 1 to 4 (Table 3A,B). This was seen most notably for nausea (≥4: OR 2.63, ≥7: OR 2.70), pain (≥4: OR 2.65, ≥7: OR 2.91) and poor appetite (≥4: OR 2.59, ≥7: OR 3.13). Patients with vulvar/vaginal and cervical cancers had the highest odds of elevated scores, and those with vulvar/vaginal cancers had the highest odds of moderate-to-severe (OR 2.35) and severe (OR 2.62) pain across all disease sites. Lower rates of material deprivation were strongly protective against all moderate-to-severe and severe symptoms, as was less severe comorbidity. Finally, time from diagnosis was also protective against both moderate-to-severe and severe scores, with a longer time from diagnosis corresponding to lower symptom scores (Table 3A,B). Two sensitivity analyses were performed to assess the robustness of the primary findings. Restricting the analysis to patients with known stage (Supplementary Table S5A,B) and to those completing the full 18-month follow-up period (Supplementary Table S6A,B) produced results that were highly consistent with the primary analyses. The direction and magnitude of the observed associations remained largely unchanged, with only modest strengthening of the association between disease stage and symptom burden after excluding patients with missing stage information. These findings support the robustness of the primary results.

## 4. Discussion

This study evaluated 207,000 unique ESAS-r assessments from 35,559 patients with gynecologic cancers to describe the prevalence, severity and temporal trends of patient-reported symptoms in the initial 18 months after cancer diagnosis. Overall, we identified that patients reported significant symptom burden at the time of diagnosis and found that higher stage, diagnosis, and elevated comorbidity were predictive of elevated symptoms, whereas older age, higher level of material resources and longer time from diagnosis were protective. These findings are consistent with previously reported data from a large cohort of mixed cancers [13].

In our cohort, symptoms were consistently highest in the first 6 months after diagnosis, highlighting the physical and psychological strain of a new cancer diagnosis and initial treatment [12,13,21,22,23,24]. Disease site was an important variable impacting self-reported symptom burden. Patients with ovarian/fallopian tube, cervical and vulvar/vaginal cancers had the highest incidence of elevated scores. Patients with advanced ovarian cancer, making up 48.1% of our ovarian cohort, often presented with manifestations of metastatic disease, including ascites, pleural effusions and bowel obstructions, which cause significant symptom burden. However, up to 70–80% of patients respond to treatment, causing a reduction in symptomatology, as shown clearly in our data. Patients with vulva/vaginal cancer reported an even higher rate of elevated symptoms; this was most notable for significantly elevated pain scores, which may be explained by the tumor location and radical treatment paradigms that result in significant morbidity. These results were consistent in the multivariable analysis after controlling for age, which is higher in this population and otherwise protective against severe symptoms (Table S2). Patients with cervical cancer had the highest odds of elevated symptom burden in many domains, including drowsiness, poor appetite, nausea, dyspnea and fatigue (Table 3A,B). Interestingly, this was seen despite most patients having early-stage disease (65% stage 1–2). The young age of this cohort could be contributory, combined with the morbidity of curative multi-modal therapy, which is often required even at early stages. Patients with endometrium/uterine cancer had the lowest incidence of elevated scores, possibly explained by the high rate of stage 1 disease (48%) and older average age in this population. Additionally, many early uterine cancers can be treated with low-morbidity interventions. Overall, symptom burden consistently decreased within the first 6 months; however, many patients continued to report elevated symptoms from 6 to 18 months post-diagnosis (Figure S2A–H). This suggests that both disease and treatment effects can be persistent and illustrates the need for longitudinal symptom screening to identify patients who are suffering. How treatment may affect both acute and long-term symptom burden was not within the scope of this study; however, we plan to investigate this in future studies of our cohort.

Stage was also noted to be an important predictor of symptom burden, with increasing stage associated with increasing patient-reported symptoms. Logically, this can be understood, as higher-stage cancers often require more aggressive treatments and patients may also present with higher symptom burden due to metastases. This finding should be interpreted in the context of a high rate of missing stage data in our study, which may have introduced bias in our results. However, a sensitivity analysis restricting the analysis to patients with known stage (Supplementary Table S5A,B) was highly consistent with our original results.

In addition to disease site, age was an important predictor of symptoms in our model, with older patients having significantly reduced odds of elevated symptoms. This is consistent with the published literature [13,22,23,24] and may be related to factors such as reduced treatment intensity, lowered expectation of wellbeing [23], less symptom-related distress [23], and differences in coping strategies [24]. Whereas age was protective, both level of comorbidity and lower socioeconomic status were strongly predictive. Highly comorbid patients report elevated symptom scores and poor functional status at baseline, which may worsen after a cancer diagnosis [25] and lead to difficulty tolerating the stresses and side effects of cancer and its treatments [24,25,26]. Socioeconomic disparity has also been linked to both poorer cancer-specific outcomes and higher symptom burden due the interplay between factors such as limited healthcare access, poor health literacy, lack of social supports and the financial strain of cancer treatments [13,27,28,29]. Together, these results demonstrate the importance of using PRO data to create targeted supportive care interventions addressing the imbalance in suffering we have identified amongst gynecologic cancer patients.

A major strength of our study is the “real-world” quality of our results. Currently, most gynecologic cancer patients completing PROs do so in a trial setting [1,30], and data collected from patients in a standard clinical environment are limited [4]. As trial patients tend to be highly selected [31] (i.e., younger, healthier, better performance status), these data likely underestimate patients’ physical and emotional burden. Furthermore, patients in trials tend to be overwhelmingly white, which limits our understanding of the experiences of a more diverse patient group [32]. To this point, the analysis of available “real-world” PROs in gynecologic cancer reveals a significant symptom burden [4,7], which is confirmed in our study. Additionally, our large sample size of over 35,000 patients and high ESAS completion rate (59.5%), which is comparable or better than that reported in other cancer populations [12,13,21,33,34,35], further add to the strength of our data.

We also note some important limitations of our study. Significant differences were identified between those patients who responded and those who did not respond to the ESAS-r questionnaire (Table S3), and baseline ESAS-r screening to account for variations in pre-cancer symptoms is not available. These differences could have introduced a selection bias into our data. Notably, patients who died during the study period were less likely to complete the ESAS-r questionnaire. We elected to include patients who died within the study to more accurately describe a “real-world” population of patients. However, as death rates varied between response cohorts and disease site cohorts, an elevated symptom burden at the end of life may have confounded our results [5]. Alternatively, this may have introduced a survivorship bias to our data as surviving patients may have been healthier, had less aggressive disease, or responded better to treatment, making outcomes appear more favorable. However, when we restricted patients to only those who completed the full 18-month follow-up period (Supplementary Table S6A,B), we again found highly consistent results. Patients with endometrium/uterine cancers and vulvar/vaginal cancers were the least likely to respond to the ESAS-r questionnaire (Tables S3 and S4). Endometrium/uterine cancer patients also reported the lowest symptom burden, suggesting that symptoms may have been under-reported in this group. In Ontario, patients with low-grade presumed early-stage endometrial cancer may be operated on by general gynecologists outside of cancer centers, which could partially account for the lower response rate in this population. There was a high rate of missing stage data in our cohort; however, in patients with a known stage, those with higher-stage cancers were more likely to respond, which could have resulted in an overestimation of symptom burden in each disease cohort. Response rates also varied notably by year. ESAS screening was rolled out provincially in 2007, explaining the lower response rate early in our study period (2007–2009), and the low screening in 2020–2022 can likely be explained by the COVID-19 pandemic. However, important patient data from these years may have been missed. Finally, as we did not have longitudinal patient-level data at each time point, the composition of patients may have differed across time points, potentially influencing the observed symptom trajectories.

## 5. Conclusions

Overall, we found that patients with gynecologic cancer reported a high prevalence of moderate-to-severe and severe symptoms at cancer diagnosis, with a substantial proportion of patients reporting persistent symptoms at 18 months. Women with gynecologic cancers experience unique challenges not seen in other cancer types, and these data demonstrate the profound and enduring impact that cancer diagnosis and treatment can have in this diverse population. The persistent burden of symptoms, particularly fatigue and impaired wellbeing, supports the routine integration of patient-reported outcome measures into gynecologic oncology care to facilitate the early identification of patients with unmet supportive care needs. Patients identified as being at increased risk for persistent symptom burden may benefit from timely referral to supportive care services and targeted interventions aimed at symptom management. More broadly, these findings highlight the value of integrating patient-reported outcomes into routine clinical practice to provide both a population-level and patient-level understanding of the symptoms that matter most to patients, enabling the development and implementation of effective quality improvement and supportive care strategies with the goal of reducing suffering and improving quality of life.

## Figures and Tables

**Table 1:** Baseline characteristics (*N* = 35,559).

Characteristic	Result
Age at diagnosis (years, mean ± SD)	61.21 ± 13.66
Age, years (*n*, %)	
18–39	2542 (7.1%)
40–49	4220 (11.9%)
50–59	8395 (23.6%)
60–69	10,518 (29.6%)
70–79	6756 (19.0%)
80+	3128 (8.8%)
Stage (TNM) (*n*, %)	
Missing	8649 (24.3%)
1	13,595 (38.2%)
2	3474 (9.8%)
3	6713 (18.9%)
4	3128 (8.8%)
Disease site (*n*, %)	
Vulva/vagina	2209 (6.2%)
Cervix	5009 (14.1%)
Endometrium/uterus	18,672 (52.5%)
Ovary/fallopian tube	9669 (27.2%)
Material resources quintile (*n*, %)	
Missing	238 (0.7%)
1	7112 (20.0%)
2	7147 (20.1%)
3	7023 (19.8%)
4	6947 (19.5%)
5	7092 (19.9%)
Immigration status (*n*, %)	
Immigrant	4969 (14.0%)
Non-immigrant	30,590 (86.0%)
Comorbidity status (*n*, %)	
Sum ADG 0–4	2769 (7.8%)
Sum ADG 5–9	11,963 (33.6%)
Sum ADG 10–14	14,808 (41.6%)
Sum ADG 15–19	5538 (15.6%)
Sum ADG ≥ 20	481 (1.4%)
Completion of follow-up (*n*, %)	
Yes	30,274 (85.1%)
No (death)	5241 (14.7%)
No (other)	44 (0.1%)

SD = standard deviation; ADG = Aggregated Diagnosis Group.

**Table 2:** Percentage of patients reporting an ESAS score of ≥ 7 or ≥ 4 at any time during the study period by diagnosis.

**(A) ESAS score of ≥7**					
	**Overall**	**Vulva/Vagina**	**Cervix**	**Endometrium/Uterus**	**Ovary/Fallopian Tube**
Anxiety	25.4	30.9	31.1	20.9	29.8
Depression	15.8	19.7	18.6	12.5	19.7
Drowsiness	18.2	24.8	23.2	13.7	22.8
Appetite	21.4	24.5	26.1	15.0	30.8
Nausea	9.3	9.7	14.3	6.0	13.1
Pain	18.5	32.6	22.7	13.6	22.6
Dyspnea	11.7	11.5	9.5	9.1	18.1
Tiredness	33.5	41.0	38.5	26.7	42.6
Wellbeing	28.5	35.8	32.1	22.4	36.7
**(B) ESAS score of ≥4**					
	**Overall**	**Vulva/Vagina**	**Cervix**	**Endometrium/Uterus**	**Ovary/Fallopian Tube**
Anxiety	51.0	55.8	57.4	45.1	57.8
Depression	35.8	41.1	41.8	30.1	42.5
Drowsiness	40.4	49.4	48.4	33.1	48.3
Appetite	44.3	50.6	53.5	34.9	56.2
Nausea	22.2	23.4	32.7	15.5	29.4
Pain	40.3	57.8	45.5	32.0	49.5
Dyspnea	27.7	29.0	25.8	22.5	38.5
Tiredness	62.6	71.1	69.3	54.7	72.3
Wellbeing	60.2	69.5	66.2	52.5	70.0

**Table 3:** Multivariable logistic regression for odds of ESAS score ≥ 7 or ≥4.

(**A) Multivariable logistic regression for odds of ESAS score ≥ 7 (severe) [aOR (95% CI)].**									
	**Anxiety**	**Depression**	**Drowsiness**	**Appetite**	**Nausea**	**Pain**	**Dyspnea**	**Tiredness**	**Wellbeing**
**Age** **at diagnosis (per 10-year increase)**	0.88 (0.86, 0.89)	0.91(0.89, 0.93)	1.04 (1.01, 1.06)	1.09 (1.06, 1.11)	0.91 (0.88, 0.94)	0.98 (0.96, 1.01)	1.25 (1.21, 1.29)	1.03 (1.01, 1.05)	1.00 (0.98, 1.02)
**Disease stage (TNM) (reference 1)**									
Stage 2	1.03 (0.95, 1.13)	1.03 (0.92, 1.16)	1.23 (1.11, 1.37)	1.38(1.25, 1.52)	1.45 (1.26, 1.67)	1.46 (1.32, 1.62)	1.01 (0.87, 1.17)	1.20 (1.11, 1.29)	1.16 (1.07, 1.25)
Stage 3	1.25 (1.16, 1.34)	1.27 (1.16, 1.39)	1.63 (1.50, 1.77)	2.11 (1.95, 2.27)	2.05 (1.83, 2.30)	2.00 (1.84, 2.17)	1.55 (1.40, 1.73)	1.58 (1.49, 1.68)	1.50 (1.41, 1.60)
Stage 4	1.50 (1.37, 1.63)	1.52 (1.37, 1.69)	2.15 (1.95, 2.37)	3.13 (2.87, 3.42)	2.70 (2.37, 3.08)	2.91 (2.65, 3.20)	2.51 (2.24, 2.81)	2.08 (1.94, 2.24)	1.99 (1.85, 2.15)
Stage missing	1.20 (1.12, 1.29)	1.18 (1.08, 1.29)	1.52 (1.40, 1.65)	1.92 (1.77, 2.08)	1.79 (1.59, 2.02)	1.85 (1.70, 2.01)	1.64 (1.48, 1.83)	1.48 (1.39, 1.57)	1.37 (1.28, 1.46)
**Diagnosis (reference endometrium/uterus)**									
Vulva/vagina	1.46 (1.33, 1.61)	1.55 (1.38, 1.75)	1.61 (1.44, 1.79)	1.46 (1.32, 1.63)	1.39 (1.19, 1.64)	2.62 (2.38, 2.89)	1.01 (0.86, 1.18)	1.51 (1.38, 1.64)	1.52 (1.40 , 1.66)
Cervix	1.34 (1.25, 1.45)	1.40 (1.27, 1.54)	1.91 (1.75, 2.08)	2.01 (1.85, 2.17)	2.14 (1.91, 2.40)	1.88 (1.72, 2.05)	1.33 (1.18, 1.51)	1.69 (1.58, 1.81)	1.47 (1.37, 1.57)
Ovary/fallopian tube	1.03 (0.97, 1.10)	1.14 (1.06, 1.23)	1.15 (1.07, 1.23)	1.36 (1.28, 1.45)	1.30 (1.18, 1.43)	1.01 (0.94, 1.08)	1.31 (1.21, 1.42)	1.21 (1.15, 1.27)	1.18 (1.11, 1.24)
**Material resources quintile (reference 5)**									
1	0.81 (0.75, 0.87)	0.65 (0.59, 0.72)	0.65 (0.59, 0.71)	0.70 (0.65, 0.76)	0.67 (0.60, 0.76)	0.59 (0.54, 0.64)	0.64 (0.57, 0.71)	0.70 (0.66, 0.75)	0.75 (0.70, 0.81)
2	0.85 (0.79, 0.92)	0.73 (0.67, 0.80)	0.77 (0.71, 0.84)	0.76 (0.71, 0.82)	0.76 (0.68, 0.85)	0.72 (0.66, 0.78)	0.74 (0.67, 0.83)	0.80 (0.75, 0.86)	0.83 (0.78, 0.89)
3	0.87 (0.81, 0.94)	0.75 (0.68, 0.83)	0.76 (0.70, 0.83)	0.82 (0.76, 0.89)	0.76 (0.67, 0.85)	0.75 (0.69, 0.82)	0.80 (0.72, 0.89)	0.82 (0.77, 0.88)	0.86 (0.80, 0.92)
4	0.87 (0.80, 0.93)	0.79 (0.72, 0.87)	0.87 (0.80, 0.95)	0.85 (0.78, 0.91)	0.75 (0.67, 0.85)	0.83 (0.76, 0.90)	0.83 (0.74, 0.92)	0.87 (0.82, 0.93)	0.88 (0.82, 0.94)
**Immigrant (reference no)**	0.90 (0.84, 0.97)	1.29 (1.18, 1.40)	0.96 (0.89, 1.05)	1.13 (1.05, 1.22)	1.09 (0.98, 1.21)	1.31 (1.21, 1.42)	1.21 (1.09, 1.33)	1.03 (0.97, 1.10)	1.22 (1.15, 1.30)
**Diagnosis year (per 1-year increase)**	0.99 (0.98, 1.00)	0.99 (0.98, 1.00)	0.98 (0.98, 0.99)	0.94 (0.93, 0.94)	0.97 (0.96, 0.98)	0.98 (0.97, 0.99)	0.96 (0.95, 0.97)	0.98 (0.97, 0.98)	0.98 (0.97, 0.99)
**Months from diagnosis (per 6-month increase)**	0.68 (0.67, 0.70)	0.87 (0.85, 0.90)	0.87 (0.85, 0.89)	0.75 (0.73, 0.77)	0.76 (0.73, 0.79)	0.96 (0.94, 0.99)	0.93 (0.90, 0.96)	0.84 (0.82, 0.85)	0.83 (0.81, 0.85)
**Level of comorbidity (reference 0–4)**									
5–9	1.23 (1.10, 1.37)	1.24 (1.07, 1.43)	1.15 (1.01, 1.30)	1.07 (0.96, 1.19)	1.13 (0.96, 1.33)	1.20 (1.06, 1.36)	1.06 (0.91, 1.24)	1.24 (1.13, 1.36)	1.11 (1.01, 1.23)
10–14	1.64 (1.48, 1.82)	1.82 (1.59, 2.09)	1.56 (1.38, 1.76)	1.27 (1.14, 1.40)	1.57 (1.34, 1.84)	1.60 (1.41, 1.80)	1.39 (1.19, 1.62)	1.71 (1.56, 1.87)	1.44 (1.31, 1.58)
15–19	2.50 (2.23, 2.80)	2.97 (2.57, 3.44)	2.36 (2.07, 2.69)	1.58 (1.41, 1.77)	2.20 (1.84, 2.61)	2.41 (2.11, 2.75)	1.88 (1.59, 2.21)	2.55 (2.31, 2.81)	1.94 (1.75, 2.14)
≥20	3.31 (2.70, 4.06)	3.85 (3.00, 4.95)	3.70 (2.98, 4.61)	1.92 (1.55, 2.37)	3.14 (2.26, 4.36)	3.99 (3.19, 4.99)	3.02 (231, 3.95)	3.97 (3.34, 4.71)	2.69 (2.24, 3.23)
**(B) Multivariable logistic regression for odds of ESAS score ≥ 4 (moderate-to-severe) [aOR (95% CI)].**									
	**Anxiety**	**Depression**	**Drowsiness**	**Appetite**	**Nausea**	**Pain**	**Dyspnea**	**Tiredness**	**Wellbeing**
**Age at diagnosis (per 10-year in-crease)**	0.88 (0.87, 0.89)	0.91 (0.89, 0.92)	0.99 (0.98, 1.01)	1.05 (1.03, 1.06)	0.88 (0.87, 0.90)	0.97 (0.95, 0.98)	1.16 (1.14, 1.19)	1.00 (0.98, 1.01)	0.97 (0.95, 0.98)
**Disease stage (TNM) (reference 1)**									
Stage 2	1.07 (1.00, 1.14)	1.13 (1.05, 1.22)	1.22 (1.14, 1.31)	1.32 (1.23, 1.41)	1.56 (1.43, 1.71)	1.36 (1.26, 1.45)	1.09 (1.00, 1.19)	1.21 (1.14, 1.28)	1.22 (1.15, 1.30)
Stage 3	1.30 (1.23, 1.37)	1.40 (1.32, 1.48)	1.59 (1.51, 1.68)	1.88 (1.79, 1.99)	2.02 (1.88, 2.17)	1.86 (1.76, 1.97)	1.51 (1.41, 1.61)	1.60 (1.52, 1.67)	1.60 (1.53, 1.68)
Stage 4	1.56 (1.46, 1.66)	1.73 (1.61, 1.87)	2.06 (1.93, 2.21)	2.59 (2.43, 2.76)	2.63 (2.41, 2.87)	2.65 (2.48, 2.84)	2.22 (2.05, 2.40)	2.03 (1.90, 2.15)	2.15 (2.02, 2.29)
Stage missing	1.22 (1.16, 1.29)	1.28 (1.21, 1.36)	1.35 (1.28, 1.43)	1.62 (1.53, 1.71)	1.68 (1.55, 1.81)	1.58 (1.49, 1.67)	1.44 (1.35, 1.54)	1.35 (1.28, 1.41)	1.38 (1.31, 1.44)
**Diagnosis (reference endometrium/uterus)**									
Vulva/Vagina	1.28 (1.18, 1.38)	1.34 (1.23, 1.46)	1.56 (1.44, 1.68)	1.48 (1.37, 1.60)	1.40 (1.26, 1.56)	2.35 (2.19, 2.53)	1.12 (1.01, 1.23)	1.45 (1.35, 1.55)	1.50 (1.40, 1.61)
Cervix	1.25 (1.18, 1.32)	1.34 (1.26, 1.43)	1.71 (1.61, 1.81)	1.92 (1.81, 2.03)	1.97 (1.82, 2.12)	1.62 (1.53, 1.73)	1.34 (1.24, 1.45)	1.58 (1.49, 1.66)	1.47 (1.39, 1.55)
Ovary/Fallopian Tube	1.03 (0.99, 1.08)	1.08 (1.03, 1.14)	1.15 (1.10, 1.21)	1.27 (1.21, 1.33)	1.27 (1.19, 1.35)	1.12 (1.07, 1.18)	1.31 (1.23, 1.38)	1.17 (1.13, 1.22)	1.17 (1.12, 1.22)
**Material resources quintile (reference 5)**									
1	0.86 (0.81, 0.91)	0.71 (0.67, 0.76)	0.72 (0.68, 0.77)	0.75 (0.71, 0.79)	0.72 (0.66, 0.77)	0.63 (0.60, 0.67)	0.67 (0.63, 0.73)	0.75 (0.71, 0.79)	0.81 (0.77, 0.85)
2	0.90 (0.85, 0.95)	0.78 (0.73, 0.83)	0.79 (0.75, 0.84)	0.80 (0.76, 0.85)	0.79 (0.73, 0.86)	0.73 (0.69, 0.78)	0.73 (0.68, 0.79)	0.80 (0.76, 0.84)	0.84 (0.80, 0.89)
3	0.90 (0.85, 0.95)	0.81 (0.76, 0.86)	0.83 (0.78, 0.88)	0.85 (0.80, 0.90)	0.80 (0.74, 0.87)	0.79 (0.74, 0.84)	0.83 (0.77, 0.89)	0.85 (0.80, 0.89)	0.86 (0.81, 0.91)
4	0.88 (0.84, 0.94)	0.80 (0.75, 0.86)	0.88 (0.82, 0.93)	0.87 (0.82, 0.92)	0.81 (0.75, 0.87)	0.83 (0.78, 0.88)	0.81 (0.75, 0.87)	0.89 (0.85, 0.94)	0.87 (0.83, 0.92)
**Immigrant (reference no)**	0.85 (0.81, 0.90)	1.22 (1.15, 1.30)	0.93 (0.87, 0.98)	1.05 (0.99, 1.11)	1.10 (1.02, 1.18)	1.36 (1.29, 1.44)	1.18 (1.11, 1.27)	0.98 (0.93, 1.03)	1.13 (1.08, 1.19)
**Diagnosis year (per 1-year increase)**	0.99 (0.98, 0.99)	0.99 (0.98, 1.00)	0.99 (0.99, 1.00)	0.93 (0.92, 0.93)	0.98 (0.97, 0.99)	0.99 (0.98, 0.99)	0.97 (0.97, 0.98)	0.98 (0.97, 0.98)	0.98 (0.98, 0.99)
**Months from diagnosis (per 6-month increase)**	0.72 (0.71, 0.73)	0.89 (0.88, 0.91)	0.87 (0.85, 0.88)	0.74 (0.72, 0.75)	0.81 (0.79, 0.83)	0.93 (0.91, 0.94)	0.95 (0.93, 0.97)	0.81 (0.80, 0.82)	0.80 (0.79, 0.81)
**Level of comorbidity (reference 0–4)**									
5–9	1.23 (1.14, 1.32)	1.29 (1.18, 1.41)	1.15 (1.06, 1.25)	1.15 (1.06, 1.24)	1.17 (1.05, 1.30)	1.19 (1.09, 1.29)	1.16 (1.05, 1.29)	1.24 (1.16, 1.33)	1.17 (1.09, 1.25)
10–14	1.66 (1.54, 1.79)	1.85 (1.69, 2.03)	1.58 (1.46, 1.72)	1.41 (1.31, 1.53)	1.51 (1.36, 1.68)	1.62 (1.49, 1.76)	1.54 (1.39, 1.71)	1.74 (1.63, 1.86)	1.63 (1.52, 1.74)
15–19	2.40 (2.21, 2.60)	2.94 (2.66, 3.24)	2.35 (2.15, 2.56)	1.84 (1.69, 2.00)	2.14 (1.91, 2.40)	2.50 (2.28, 2.73)	2.18 (1.95, 2.43)	2.64 (2.44, 2.85)	2.38 (2.21, 2.57)
≥20	3.06 (2.60, 3.60)	3.96 (3.32, 4.74)	3.69 (3.12, 4.35)	2.45 (2.08, 2.88)	3.21 (2.58, 4.00)	4.40 (3.72, 5.19)	3.30 (2.71, 4.01)	4.75 (4.01, 5.64)	3.61 (3.09, 4.23)

## Data Availability

The raw data supporting the conclusions of this article will be made available by the authors on request.

## Supplementary Materials

The following supporting information can be downloaded at: https://www.mdpi.com/article/10.3390/cancers18162628/s1, Figure S1: Edmonton Symptom Assessment Scale-revised (ESAS-r); Figure S2: (A–H) Percentage of patients reporting moderate-to-severe (≥4) or severe scores (≥7) on ESAS symptoms by month from diagnosis and by diagnosis group; Table S1: ICD-O3 codes; Table S2: Baseline characteristics by diagnosis (*n* = 35,559); Table S3: Baseline characteristics by ESAS completion (*N* = 59,715); Table S4: Patients completing ≥1 ESAS by month since diagnosis (*N* = 35,559); Table S5: (A,B) Sensitivity analysis (restricted to known stage). Multivariable logistic regression for odds of ESAS score ≥ 7 or ≥4; Table S6: (A,B) Sensitivity analysis (restricted to follow-up 18 m). Multivariable logistic regression for odds of ESAS score ≥ 7 or ≥4.
