# Supplementary material for: Patient-Reported Symptom Burden and Temporal Trends During the First 18 Months After Gynecologic Cancer Diagnosis: A Population-Based Study
Source: Cancers (Basel). 2026 Aug 14;18(16):2628. doi: 10.3390/cancers18162628 (PMC13510903; doi:10.3390/cancers18162628)
Supplement: Supplementary file 1 [file cancers-18-02628-s001.zip › cancers-4409784-supplementary.pdf]

Figure S1. Edmonton Symptom Assessment Scale-revised (ESAS-r).

Please circle the number that best describes how you feel NOW:

No Pain	0	1	2	3	4	5	6	7	8	9	10	Worst Possible Pain
No Tiredness (Tiredness = lack of energy)	0	1	2	3	4	5	6	7	8	9	10	Worst Possible Tiredness
No Drowsiness (Drowsiness = feeling sleepy)	0	1	2	3	4	5	6	7	8	9	10	Worse Possible Drowsiness
No Nausea	0	1	2	3	4	5	6	7	8	9	10	Worst Possible Nausea
No Lack of Appetite	0	1	2	3	4	5	6	7	8	9	10	Worst Possible Lack of Appetite
No Shortness of Breath	0	1	2	3	4	5	6	7	8	9	10	Worst Possible Shortness of Breath
No Depression (Depression = feeling sad)	0	1	2	3	4	5	6	7	8	9	10	Worse Possible Depression
No Anxiety (Anxiety = feeling nervous)	0	1	2	3	4	5	6	7	8	9	10	Worst Possible Anxiety
Best Wellbeing (Wellbeing = how you feel overall)	0	1	2	3	4	5	6	7	8	9	10	Worst Possible Wellbeing
No _________ Other Problem (ex: constipation)	0	1	2	3	4	5	6	7	8	9	10	Worst Possible _________

Figure S2. (A–H) Percentage of patients reporting moderate-to-severe (≥4) or severe scores (≥7) on ESAS symptoms by month from diagnosis and by diagnosis group.

Figure S2A. Percentage of vulva/vagina patients reporting moderate-to-severe scores (ESAS>=4) by month from diagnosis (n = 2,209).

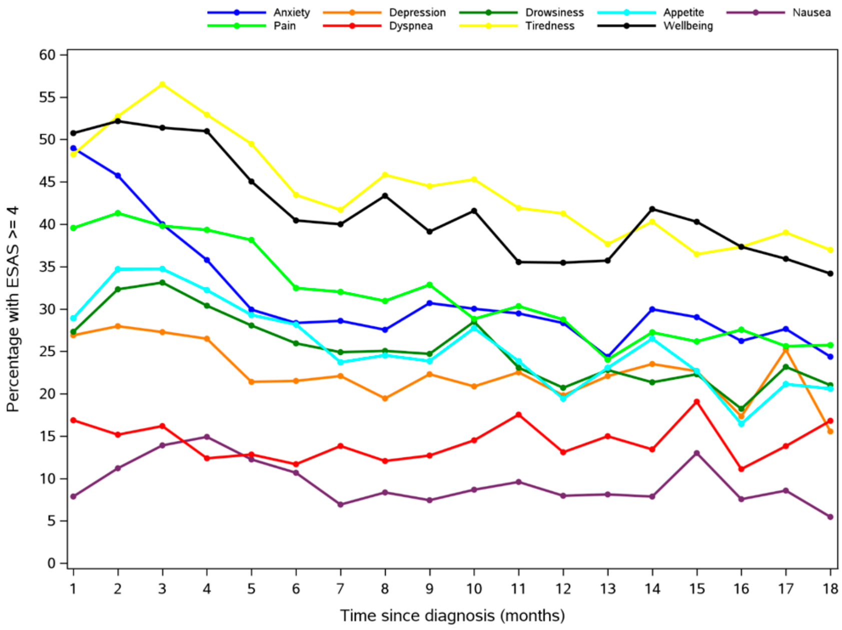

Figure S2B. Percentage of vulva/vagina patients reporting severe scores (ESAS>=7) by month from diagnosis (n = 2,209)

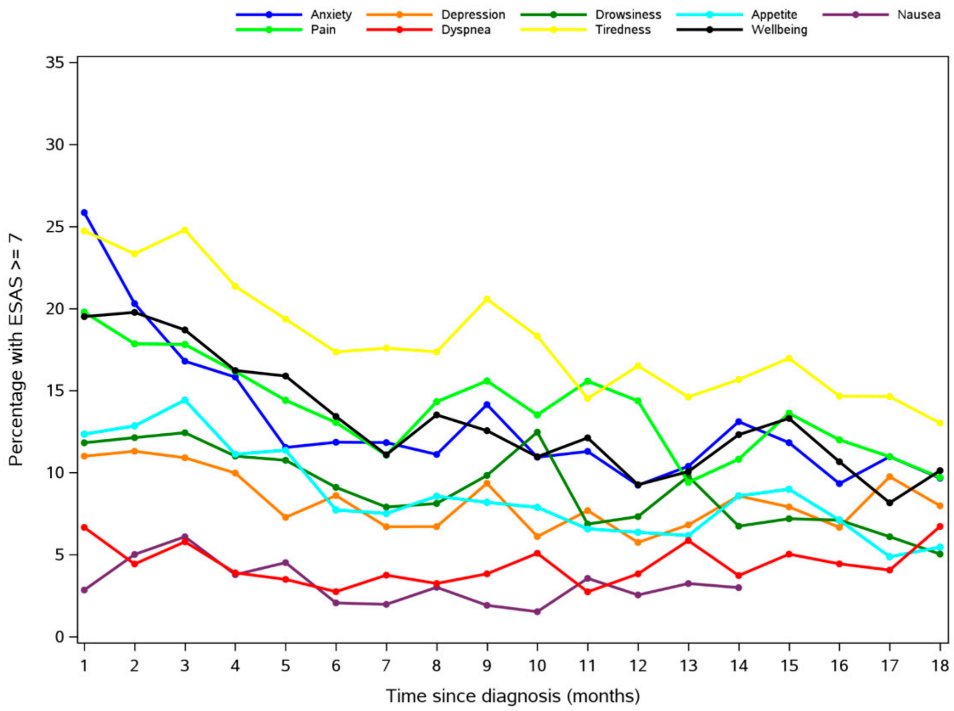

Figure S2C. Percentage of cervix patients reporting moderate-to-severe scores (ESAS>=4) by month from diagnosis (n = 5,009)

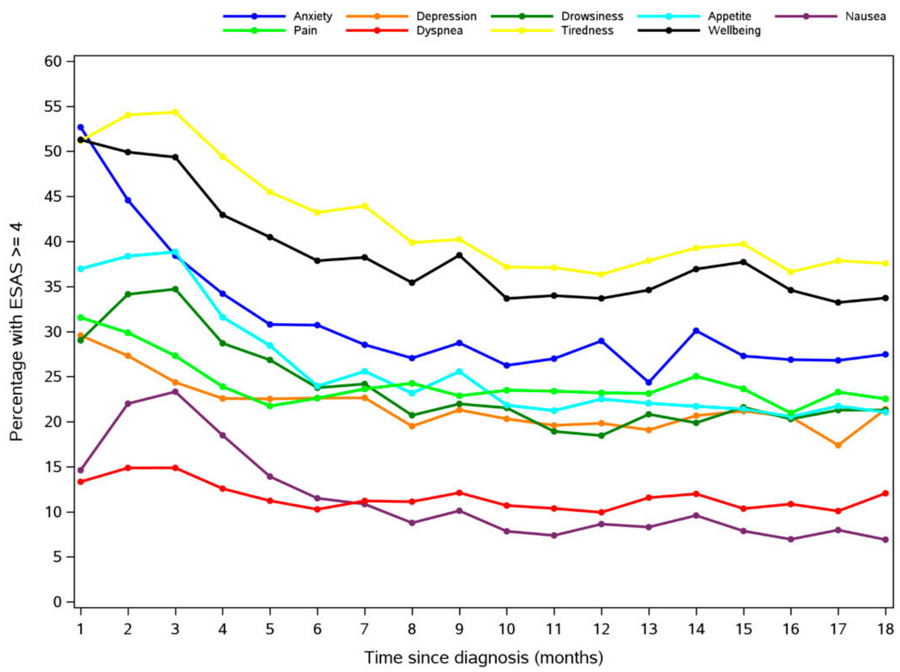

Figure S2D. Percentage of cervix patients reporting severe scores (ESAS>=7) by month from diagnosis (n = 5,009)

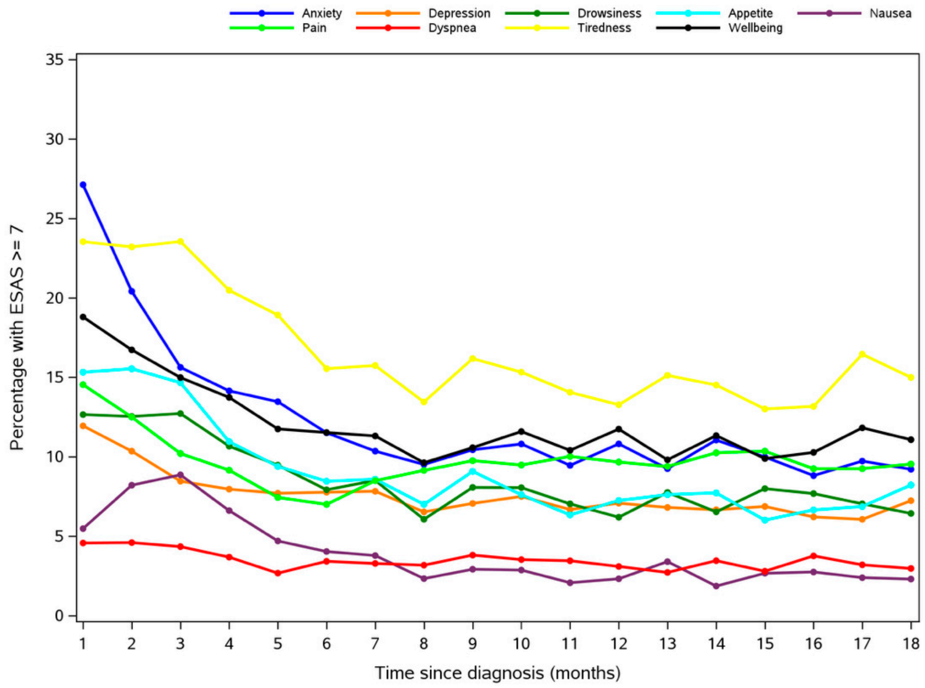

Figure S2E. Percentage of endometrium/uterus patients reporting moderate-to-severe scores (ESAS>=4) by month from diagnosis (n = 18,672)

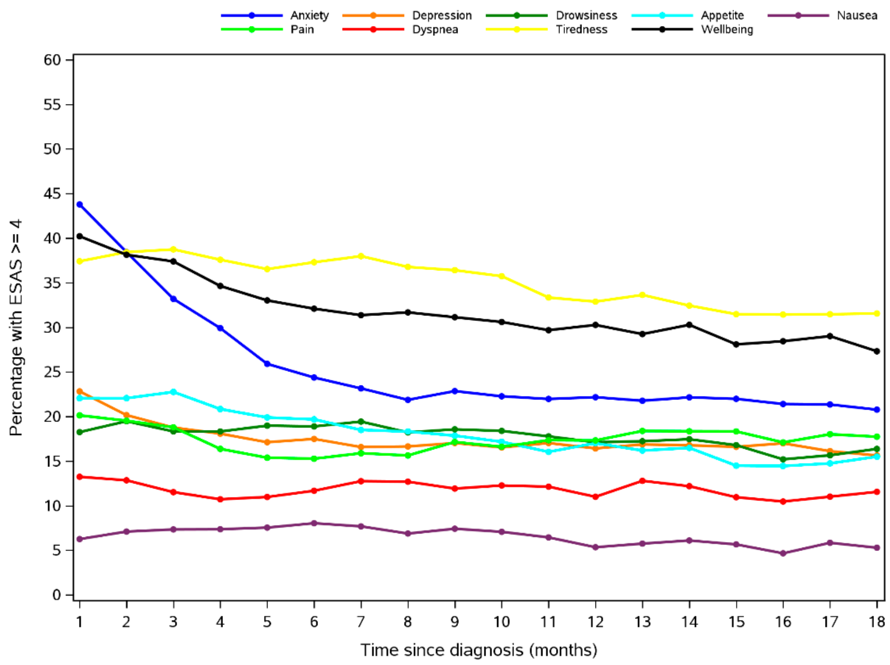

Figure S2F. Percentage of endometrium/uterus patients reporting severe scores (ESAS>=7) by month from diagnosis (n = 18,672)

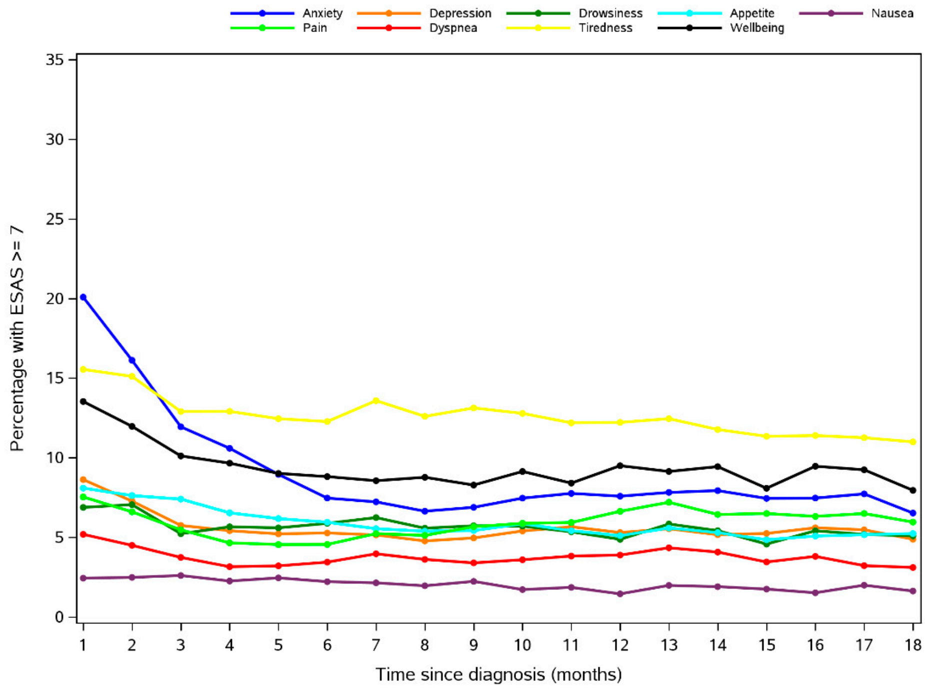

Figure S2G. Percentage of ovary/fallopian tube patients reporting moderate-to-severe scores (ESAS>=4) by month from diagnosis (n = 9,669)

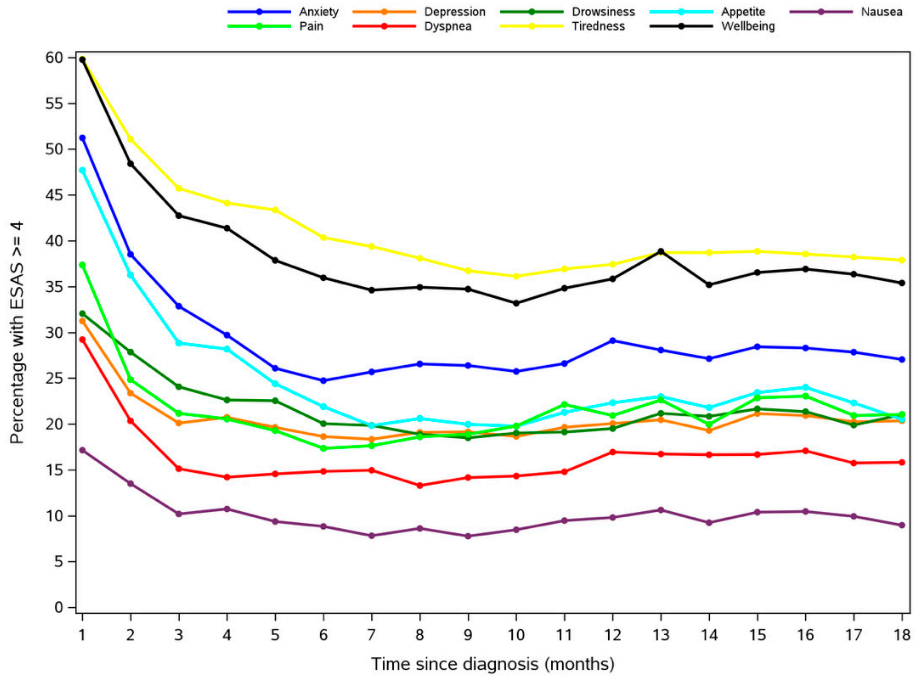

Figure S2H. Percentage of ovary/fallopian tube patients reporting severe scores (ESAS>=7) by month from diagnosis (n = 9,669)

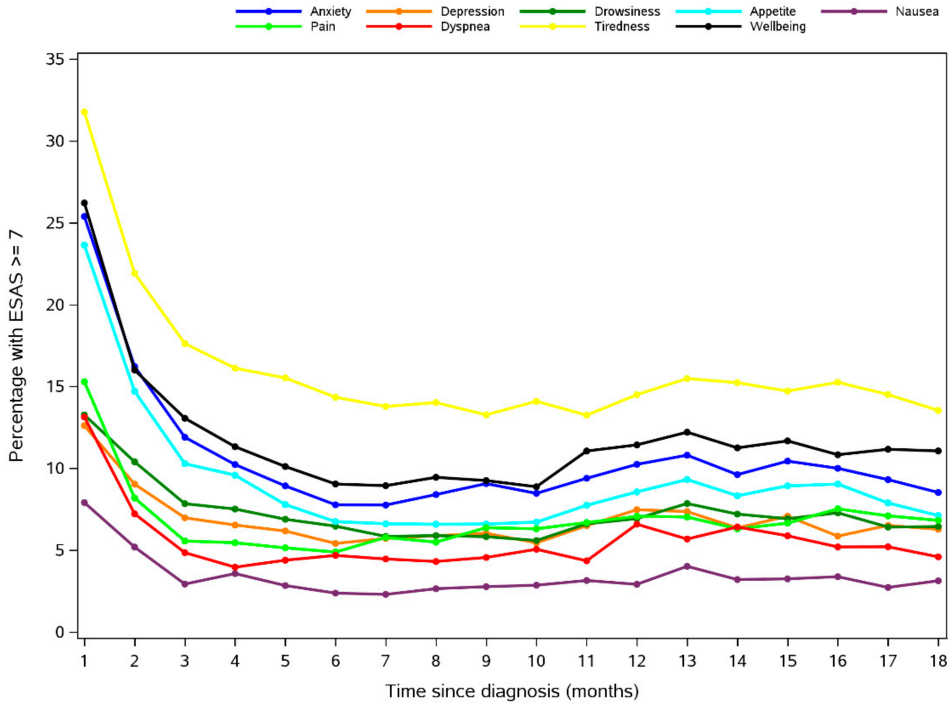

Table S1. ICD-O3 codes.

Site	ICD-03 code
Vulva / Vagina	C51.0, C51.1, C51.2, C51.8, C51.9, C52.9
Cervix	C53.0, C53.1, C53.8, C53.9
Endometrium/Uterus	C54.0, C54.1, C54.2, C54.3, C54.8, C54.9, C55.9
Ovary/Fallopian tube	C56.9, C57.0, C57.1, C57.2, C57.3, C57.4

Table S2. Baseline characteristics by diagnosis (n = 35,559).

Characteristic	Vulva/Vagina N = 2,209	Cervix N=5,009	Endometrium/ Uterus N=18,672	Ovary/Fallopian Tube N = 9,669	P-value
Age at diagnosis (years, mean +/- SD)	66.52 ± 14.45	49.96 ± 14.69	63.64 ± 11.31	61.16 ± 14.01	<0.001
Age, years (n, %)					<0.001
18-39	73 (3.3%)	1,353 (27.0%)	462 (2.5%)	654 (6.8%)	
40-49	216 (9.8%)	1,397 (27.9%)	1,384 (7.4%)	1,223 (12.6%)	
50-59	425 (19.2%)	1,009 (20.1%)	4,666 (25.0%)	2,295 (23.7%)	
60-69	530 (24.0%)	695 (13.9%)	6,632 (35.5%)	2,661 (27.5%)	
70-79	489 (22.1%)	346 (6.9%)	3,956 (21.2%)	1,965 (20.3%)	
80+	476 (21.5%)	209 (4.2%)	1,572 (8.4%)	871 (9.0%)	
Stage (TNM) (n, %)					<0.001
Missing	637 (28.8%)	459 (9.2%)	4,817 (25.8%)	2,736 (28.3%)	
1	771 (34.9%)	2,297 (45.9%)	8,968 (48.0%)	1,559 (16.1%)	
2	228 (10.3%)	956 (19.1%)	1,571 (8.4%)	719 (7.4%)	
3	385 (17.4%)	801 (16.0%)	2,170 (11.6%)	3,357 (34.7%)	
4	188 (8.5%)	496 (9.9%)	1,146 (6.1%)	1,298 (13.4%)	
Material Resources Quintile (n,%)					<0.001
Missing	17 (0.8%)	53 (1.1%)	120 (0.6%)	48 (0.5%)	
1	406 (18.4%)	891 (17.8%)	3,756 (20.1%)	2,059 (21.3%)	
2	396 (17.9%)	935 (18.7%)	3,750 (20.1%)	2,066 (21.4%)	
3	430 (19.5%)	981 (19.6%)	3,676 (19.7%)	1,936 (20.0%)	
4	429 (19.4%)	1,008 (20.1%)	3,684 (19.7%)	1,826 (18.9%)	
5	531 (24.0%)	1,141 (22.8%)	3,686 (19.7%)	1,734 (17.9%)	
Immigration status (n,%)					<0.001
Immigrant	158 (7.2%)	808 (16.1%)	2,548 (13.6%)	1,455 (15.0%)	
Non-immigrant	2,051 (92.8%)	4,201 (83.9%)	16,124 (86.4%)	8,214 (85.0%)	
Comorbidity status (n,%)					<0.001
Sum ADG 0-4	131 (5.9%)	708 (14.1%)	1,336 (7.2%)	594 (6.1%)	
Sum ADG 5-9	574 (26.0%)	1,941 (38.8%)	6,422 (34.4%)	3,026 (31.3%)	
Sum ADG 10-14	962 (43.5%)	1,799 (35.9%)	7,814 (41.8%)	4,233 (43.8%)	
Sum ADG 15-19	486 (22.0%)	521 (10.4%)	2,849 (15.3%)	1,682 (17.4%)	
Sum ADG <=20	56 (2.5%)	40 (0.8%)	251 (1.3%)	134 (1.4%)	
Completion of follow up (n,%)					<0.001
Yes	1,704 (77.1%)	4,306 (86.0%)	16,645 (89.1%)	7,619 (78.8%)	

No (death)<br>No (other)	505 (22.9%)<br>0 (0.0%)	689 (13.8%)<br>14 (0.3%)	2,006 (10.7%)<br>21 (0.1%)	2,041 (21.1%)<br>9 (0.1%)	

Table S2 legend: SD = standard deviation, ADG = aggregated diagnosis group

Table S3. Baseline characteristics by ESAS completion (N = 59,715).

Characteristic	ESAS (N=35,559)	No ESAS (24,156)	P-value
Age at diagnosis (years, mean +/- SD)	61.21 ± 13.66	62.56 ± 15.03	<0.001
Age, years (n, %)			
18-39	2,542 (7.1%)	1,769 (7.3%)	<0.001
40-49	4,220 (11.9%)	2,592 (10.7%)	
50-59	8395 (23.6%)	5,773 (23.9%)	
60-69	10,518 (29.6%)	6,285 (26.0%)	
70-79	6,756 (19.0%)	4,252 (17.6%)	
80+	3,128 (8.8%)	3,485 (14.4%)	
Stage (TNM) (n, %)			
Missing	8,649 (24.3%)	12,467 (51.6%)	<0.001
1	13,595 (38.2%)	7,836 (32.4%)	
2	3,474 (9.8%)	934 (3.9%)	
3	6,713 (18.9%)	1,621 (6.7%)	
4	3,128 (8.8%)	1,298 (5.4%)	
Diagnosis (n,%)			
Vulva/Vagina	2,209 (6.2%)	1,634 (6.8%)	<0.001
Cervix	5,009 (14.1%)	2,746 (11.4%)	
Endometrium/Uterus	18,672 (52.5%)	14,497 (60.0%)	
Ovary/Fallopian Tube	9,669 (27.2%)	5,279 (21.9%)	
Material Resources Quintile			
(n,%)	238 (0.7%)	218 (0.9%)	<0.001
Missing	7,112 (20.0%)	4,453 (18.4%)	
1	7,147 (20.1%)	4,797 (19.9%)	
2	7,023 (19.8%)	4,830 (20.0%)	
3	6,947 (19.5%)	4,718 (19.5%)	
4	7,092 (19.9%)	5,140 (21.3%)	
5			
Immigration status (n,%)			
Immigrant	4,969 (14.0%)	3,083 (12.8%)	<0.001
Non-immigrant	30,590 (86.0%)	21,073 (87.2%)	
Comorbidity status (n,%)			
Sum ADG 0-4	2,769 (7.8%)	1,680 (7.0%)	<0.001
Sum ADG 5-9	11,963 (33.6%)	7,603 (31.5%)	
Sum ADG 10-14	14,808 (41.6%)	10,181 (42.1%)	
Sum ADG 15-19	5,538 (15.6%)	4,244 (17.6%)	
Sum ADG <=20	481 (1.4%)	448 (1.9%)	
Completion of follow up (n,%) Yes	30,274 (85.1%)	18,905 (78.3%)	<0.001

No (death)<br>No (other)	5,241 (14.7%)<br>44 (0.1%)	5,188 (21.5%)<br>63 (0.3%)	
Diagnosis year (n,%)<br>2007<br>2008<br>2009<br>2010<br>2011<br>2012<br>2013<br>2014<br>2015<br>2016<br>2017<br>2018<br>2019<br>2020<br>2021<br>2022	 707 (2.0%)<br>1,136 (3.2%)<br>1,660 (4.7%)<br>2,137 (6.0%)<br>2,414 (6.8%)<br>2,491 (7.0%)<br>2,620 (7.4%)<br>2,680 (7.5%)<br>2,837 (8.0%)<br>3,010 (8.5%)<br>3,129 (8.8%)<br>3,160 (8.9%)<br>3,044 (8.6%)<br>1,773 (5.0%)<br>2,236 (6.3%)<br>525 (1.5%)	 2,354 (9.7%)<br>1,962 (8.1%)<br>1,625 (6.7%)<br>1,270 (5.3%)<br>1,121 (4.6%)<br>1,172 (4.9%)<br>1,066 (4.4%)<br>1,042 (4.3%)<br>1,143 (4.7%)<br>1,233 (5.1%)<br>1,212 (5.0%)<br>1,336 (5.5%)<br>1,589 (6.6%)<br>2,670 (11.1%)<br>2,623 (10.9%)<br>738 (3.1%)	<0.001

Table S3 legend: SD = standard deviation, ADG = aggregated diagnosis group

Table S4. Patients completing ≥1 ESAS by month since diagnosis (N = 35,559).

Months since diagnosis	Overall (n,%)	Vulva/Vagina (n,%)	Cervix (n,%)	Endometrium/ Uterus (n,%)	Ovary/Fallopian Tube (n,%)
1	10500 (29.5)	740 (33.5)	1535 (30.6)	4515 (24.2)	3710 (38.4)
2	12524 (35.3)	858 (39.0)	2242 (44.8)	4916 (26.3)	4508 (46.8)
3	13749 (39.0)	936 (42.6)	2235 (44.9)	6080 (32.7)	4498 (47.3)
4	13632 (38.9)	873 (40.1)	1848 (37.3)	6598 (35.7)	4313 (45.8)
5	12410 (35.7)	688 (32.0)	1533 (31.1)	5829 (31.7)	4360 (46.9)
6	11210 (32.5)	582 (27.5)	1315 (26.9)	5318 (29.1)	3995 (43.5)
7	10029 (29.4)	507 (24.3)	1189 (24.5)	4992 (27.5)	3341 (36.8)
8	8346 (24.6)	434 (21.0)	1072 (22.2)	4238 (23.5)	2602 (28.9)
9	7326 (21.8)	418 (20.6)	892 (18.7)	3709 (20.7)	2307 (26.0)
10	6911 (20.8)	393 (19.7)	908 (19.2)	3372 (19.0)	2238 (25.4)
11	6235 (18.9)	367 (18.7)	868 (18.6)	2904 (16.5)	2096 (24.1)
12	5664 (17.4)	315 (16.5)	778 (16.9)	2550 (14.5)	2021 (23.6)
13	5559 (17.2)	308 (16.4)	735 (16.1)	2420 (13.9)	2096 (24.9)
14	5410 (17.0)	268 (14.5)	751 (16.6)	2362 (13.7)	2029 (24.5)
15	5165 (16.4)	279 (15.4)	716 (16.0)	2232 (13.0)	1938 (23.8)
16	5007 (16.0)	225 (12.6)	692 (15.6)	2109 (12.4)	1981 (24.7)
17	4734 (15.3)	246 (14.0)	627 (14.3)	1957 (11.6)	1904 (24.1)
18	4665 (15.3)	238 (13.8)	608 (14.0)	2031 (12.1)	1788 (23.1)

Table S5. (A,B) Sensitivity Analysis (Restricted to known stage). Multivariable logistic regression for odds of ESAS score ≥ 7 or ≥4.

Table S5A. Multivariable logistic regression for odds of ESAS score >=7 (severe) [aOR (95% CI)].

	Anxiety	Depress-ion	Drowsi-ness	Appetite	Nausea	Pain	Dyspnea	Tired-ness	Well-being
**Age at diagnosis (per 10-year increase)**	0.86 (0.84 , 0.88)	0.89 (0.86 , 0.91)	1.01 (0.98 , 1.04)	1.05 (1.02 , 1.08)	0.89 (0.86 , 0.92)	0.97 (0.94 , 0.99)	1.22 (1.18 , 1.27)	1.00 (0.98 , 1.03)	0.98 (0.96 , 1.01)
**Disease stage (TNM) (reference 1)**									
Stage 2	1.04 (0.95 , 1.13)	1.04 (0.93 , 1.17)	1.25 (1.13 , 1.39)	1.41 (1.28 , 1.55)	1.48 (1.29 , 1.70)	1.48 (1.34 , 1.65)	1.02 (0.88 , 1.19)	1.22 (1.13 , 1.32)	1.17 (1.08 , 1.27)
Stage 3	1.25 (1.17 , 1.35)	1.31 (1.19 , 1.43)	1.69 (1.55 , 1.83)	2.20 (2.04 , 2.38)	2.12 (1.89 , 2.38)	2.07 (1.91 , 2.25)	1.61 (1.45 , 1.79)	1.64 (1.54 , 1.74)	1.53 (1.44 , 1.64)
Stage 4	1.51 (1.39 , 1.64)	1.57 (1.41 , 1.74)	2.22 (2.02 , 2.45)	3.27 (2.99 , 3.57)	2.80 (2.45 , 3.19)	3.01 (2.73 , 3.31)	2.61 (2.32 , 2.93)	2.16 (2.00 , 2.32)	2.04 (1.89 , 2.20)
**Diagnosis (reference endometrium/ uterus)**									
Vulva/Vagina	1.45 (1.29 , 1.62)	1.46 (1.27 , 1.69)	1.62 (1.43 , 1.84)	1.41 (1.24 , 1.60)	1.26 (1.04 , 1.52)	2.66 (2.38 , 2.98)	0.98 (0.81 , 1.18)	1.48 (1.34 , 1.63)	1.43 (1.29 , 1.58)
Cervix	1.33 (1.23 , 1.45)	1.37 (1.23 , 1.52)	1.84 (1.67 , 2.02)	1.89 (1.74 , 2.06)	2.03 (1.79 , 2.30)	1.78 (1.62 , 1.95)	1.29 (1.13 , 1.48)	1.59 (1.48 , 1.71)	1.42 (1.32 , 1.54)
Ovary/Fallo-pian Tube	1.04 (0.97 , 1.11)	1.08 (0.99 , 1.19)	1.08 (0.99 , 1.17)	1.24 (1.15 , 1.33)	1.21 (1.08 , 1.36)	0.92 (0.85 , 1.00)	1.21 (1.10 , 1.33)	1.12 (1.05 , 1.19)	1.12 (1.05 , 1.19)
**Material Resources Quintile (reference 5)**									
1	0.83 (0.76 , 0.90)	0.67 (0.60 , 0.74)	0.63 (0.57 , 0.70)	0.70 (0.64 , 0.76)	0.69 (0.61 , 0.79)	0.58 (0.52 , 0.64)	0.63 (0.55 , 0.71)	0.71 (0.66 , 0.76)	0.75 (0.69 , 0.81)
2	0.89 (0.82 , 0.97)	0.74 (0.67 , 0.83)	0.74 (0.67 , 0.82)	0.75 (0.69 , 0.82)	0.79 (0.69 , 0.90)	0.70 (0.63 , 0.77)	0.76 (0.67 , 0.86)	0.81 (0.75 , 0.87)	0.85 (0.79 , 0.92)
3	0.86 (0.79 , 0.94)	0.75 (0.67 , 0.83)	0.72 (0.65 , 0.80)	0.78 (0.72 , 0.85)	0.77 (0.67 , 0.88)	0.71 (0.64 , 0.78)	0.79 (0.69 , 0.89)	0.80 (0.74 , 0.86)	0.81 (0.75 , 0.88)
4	0.87 (0.80 , 0.95)	0.79 (0.71 , 0.88)	0.85 (0.77 , 0.93)	0.85 (0.78 , 0.93)	0.77 (0.68 , 0.88)	0.81 (0.74 , 0.89)	0.87 (0.77 , 0.99)	0.88 (0.82 , 0.94)	0.89 (0.82 , 0.96)

Immigrant (reference no)	0.91 (0.83 , 0.99)	1.29 (1.17 , 1.43)	0.92 (0.84 , 1.02)	1.12 (1.03 , 1.22)	1.12 (0.99 , 1.26)	1.30 (1.19 , 1.43)	1.13 (1.01 , 1.28)	1.01 (0.94 , 1.09)	1.21 (1.12 , 1.30)
Diagnosis year (per 1 year increase)	0.99 (0.99 , 1.00)	0.99 (0.98 , 1.00)	0.99 (0.98 , 1.00)	0.93 (0.93 , 0.94)	0.97 (0.96 , 0.98)	0.98 (0.97 , 0.99)	0.96 (0.95 , 0.97)	0.98 (0.97 , 0.99)	0.98 (0.98 , 0.99)
Months from diagnosis (per 6-month increase)	0.68 (0.66 , 0.70)	0.87 (0.85 , 0.90)	0.88 (0.85 , 0.90)	0.75 (0.73 , 0.77)	0.76 (0.72 , 0.79)	0.97 (0.94 , 1.00)	0.95 (0.91 , 0.98)	0.84 (0.82 , 0.86)	0.83 (0.81 , 0.86)
Level of comorbidity (reference 0-4)									
5-9	1.21 (1.07 , 1.36)	1.20 (1.02 , 1.40)	1.15 (1.00 , 1.33)	1.08 (0.96 , 1.22)	1.08 (0.90 , 1.28)	1.20 (1.04 , 1.38)	1.08 (0.90 , 1.30)	1.27 (1.15 , 1.41)	1.11 (1.00 , 1.24)
10-14	1.68 (1.49 , 1.89)	1.81 (1.55 , 2.11)	1.64 (1.43 , 1.88)	1.33 (1.19 , 1.50)	1.57 (1.32 , 1.86)	1.60 (1.39 , 1.84)	1.47 (1.23 , 1.76)	1.82 (1.64 , 2.02)	1.49 (1.34 , 1.66)
15-19	2.53 (2.23 , 2.87)	2.89 (2.46 , 3.41)	2.50 (2.15 , 2.90)	1.70 (1.50 , 1.94)	2.23 (1.84 , 2.70)	2.52 (2.17 , 2.94)	2.02 (1.67 , 2.44)	2.73 (2.44 , 3.05)	2.05 (1.83 , 2.30)
≥20	3.32 (2.62 , 4.22)	3.77 (2.81 , 5.06)	4.17 (3.23 , 5.37)	2.00 (1.57 , 2.56)	3.19 (2.17 , 4.69)	3.75 (2.88 , 4.88)	3.07 (2.21 , 4.28)	4.06 (3.32 , 4.97)	2.74 (2.21 , 3.40)

Table S5B. Multivariable logistic regression for odds of ESAS score >=4 (moderate-to-severe) [aOR (95% CI)].

	Anxiety	Depress-ion	Drowsi-ness	Appetite	Nausea	Pain	Dyspnea	Tired-ness	Well-being
**Age at diagnosis (per 10-year increase)**	0.87 (0.85 , 0.88)	0.89 (0.87 , 0.91)	0.98 (0.96 , 0.99)	1.02 (1.00 , 1.04)	0.86 (0.84 , 0.88)	0.95 (0.93 , 0.96)	1.14 (1.11 , 1.16)	0.97 (0.96 , 0.99)	0.95 (0.93 , 0.96)
**Disease stage (TNM) (reference 1)**									
Stage 2	1.07 (1.01 , 1.14)	1.14 (1.06 , 1.23)	1.23 (1.15 , 1.32)	1.35 (1.26 , 1.44)	1.60 (1.46 , 1.75)	1.38 (1.29 , 1.48)	1.11 (1.01 , 1.21)	1.22 (1.15 , 1.30)	1.24 (1.17 , 1.31)
Stage 3	1.31 (1.24 , 1.38)	1.42 (1.33 , 1.51)	1.63 (1.54 , 1.73)	1.96 (1.86 , 2.07)	2.10 (1.95 , 2.26)	1.95 (1.84 , 2.06)	1.55 (1.44 , 1.66)	1.64 (1.56 , 1.72)	1.64 (1.56 , 1.72)
Stage 4	1.56 (1.46 , 1.67)	1.75 (1.63 , 1.89)	2.11 (1.97 , 2.26)	2.69 (2.52 , 2.87)	2.72 (2.49 , 2.97)	2.76 (2.58 , 2.96)	2.28 (2.10 , 2.47)	2.07 (1.95 , 2.21)	2.20 (2.06 , 2.34)
**Diagnosis (reference endometrium/ uterus)**									
Vulva/Vagina	1.33 (1.22 , 1.45)	1.36 (1.23 , 1.50)	1.58 (1.44 , 1.73)	1.45 (1.33 , 1.59)	1.36 (1.20 , 1.54)	2.39 (2.19 , 2.60)	1.08 (0.96 , 1.22)	1.43 (1.32 , 1.55)	1.49 (1.37 , 1.61)
Cervix	1.23 (1.16 , 1.31)	1.31 (1.22 , 1.41)	1.64 (1.53 , 1.75)	1.78 (1.67 , 1.89)	1.84 (1.69 , 1.99)	1.53 (1.43 , 1.64)	1.29 (1.18 , 1.40)	1.50 (1.41 , 1.58)	1.40 (1.33 , 1.49)
Ovary/Fallo-pian Tube	1.02 (0.97 , 1.08)	1.06 (1.00 , 1.13)	1.09 (1.03 , 1.15)	1.15 (1.09 , 1.21)	1.16 (1.08 , 1.25)	1.01 (0.96 , 1.07)	1.24 (1.16 , 1.32)	1.11 (1.06 , 1.17)	1.12 (1.07 , 1.18)
**Material Resources Quintile (reference 5)**									
1	0.85 (0.80 , 0.91)	0.72 (0.67 , 0.78)	0.72 (0.67 , 0.77)	0.75 (0.70 , 0.80)	0.72 (0.66 , 0.78)	0.62 (0.58 , 0.67)	0.66 (0.61 , 0.72)	0.74 (0.70 , 0.79)	0.81 (0.76 , 0.86)
2	0.91 (0.86 , 0.97)	0.78 (0.73 , 0.84)	0.79 (0.74 , 0.84)	0.81 (0.76 , 0.86)	0.80 (0.74 , 0.88)	0.72 (0.68 , 0.77)	0.75 (0.69 , 0.81)	0.79 (0.75 , 0.84)	0.85 (0.80 , 0.90)
3	0.88 (0.82 , 0.93)	0.78 (0.73 , 0.84)	0.79 (0.74 , 0.85)	0.83 (0.77 , 0.88)	0.79 (0.72 , 0.87)	0.75 (0.70 , 0.81)	0.80 (0.74 , 0.87)	0.82 (0.77 , 0.87)	0.83 (0.78 , 0.88)
4	0.88 (0.82 , 0.94)	0.79 (0.74 , 0.85)	0.85 (0.80 , 0.91)	0.86 (0.81 , 0.92)	0.81 (0.74 , 0.88)	0.82 (0.76 , 0.87)	0.82 (0.76 , 0.89)	0.88 (0.83 , 0.94)	0.86 (0.81 , 0.92)
**Immigrant (reference no)**	0.87 (0.81 , 0.92)	1.22 (1.13 , 1.30)	0.92 (0.86 , 0.99)	1.05 (0.99 , 1.12)	1.13 (1.04 , 1.22)	1.37 (1.28 , 1.46)	1.13 (1.04 , 1.22)	0.97 (0.92 , 1.03)	1.13 (1.07 , 1.20)

**Diagnosis year (per 1 year increase)**	0.99 (0.99 , 1.00)	0.99 (0.99 , 1.00)	0.99 (0.99 , 1.00)	0.92 (0.92 , 0.93)	0.98 (0.97 , 0.99)	0.99 (0.98 , 0.99)	0.97 (0.97 , 0.98)	0.98 (0.97 , 0.98)	0.99 (0.98 , 0.99)
**Months from diagnosis (per 6-month increase)**	0.72 (0.71 , 0.73)	0.89 (0.87 , 0.91)	0.87 (0.85 , 0.88)	0.73 (0.71 , 0.74)	0.81 (0.78 , 0.83)	0.93 (0.91 , 0.95)	0.95 (0.93 , 0.97)	0.80 (0.79 , 0.82)	0.79 (0.78 , 0.81)
**Level of comorbidity (reference 0-4)**									
5-9	1.20 (1.11 , 1.31)	1.28 (1.15 , 1.42)	1.17 (1.07 , 1.29)	1.17 (1.08 , 1.28)	1.16 (1.03 , 1.30)	1.21 (1.10 , 1.33)	1.20 (1.07 , 1.35)	1.24 (1.15 , 1.34)	1.17 (1.08 , 1.26)
10-14	1.65 (1.52 , 1.80)	1.85 (1.68 , 2.05)	1.63 (1.49 , 1.79)	1.48 (1.36 , 1.61)	1.51 (1.35 , 1.70)	1.67 (1.52 , 1.84)	1.63 (1.45 , 1.83)	1.77 (1.64 , 1.91)	1.67 (1.55 , 1.80)
15-19	2.39 (2.18 , 2.63)	2.92 (2.62 , 3.26)	2.43 (2.20 , 2.69)	1.94 (1.76 , 2.13)	2.17 (1.91 , 2.47)	2.62 (2.37 , 2.90)	2.28 (2.01 , 2.58)	2.72 (2.50 , 2.97)	2.44 (2.24 , 2.66)
≥20	3.05 (2.52 , 3.71)	3.90 (3.16 , 4.81)	4.01 (3.30 , 4.87)	2.58 (2.13 , 3.12)	3.44 (2.66 , 4.44)	4.52 (3.73 , 5.48)	3.52 (2.80 , 4.42)	4.88 (4.00 , 5.95)	3.72 (3.10 , 4.47)

Table S6. (A,B) Sensitivity analysis (restricted to follow-up 18 m). Multivariable logistic regression for odds of ESAS score ≥ 7 or ≥4.

Table S6A. Multivariable logistic regression for odds of ESAS score >=7 (severe) [aOR (95% CI)].

	Anxiety	Depression	Drowsiness	Appetite	Nausea	Pain	Dyspnea	Tiredness	Well-being
**Age at diagnosis (per 10-year increase)**	0.84 (0.82 , 0.86)	0.86 (0.83 , 0.88)	0.95 (0.92 , 0.98)	1.01 (0.98 , 1.04)	0.85 (0.82 , 0.89)	0.95 (0.92 , 0.98)	1.17 (1.13 , 1.22)	0.95 (0.93 , 0.97)	0.94 (0.92 , 0.96)
**Disease stage (TNM) (reference 1)**									
Stage 2	1.02 (0.93 , 1.12)	1.01 (0.89 , 1.14)	1.19 (1.06 , 1.33)	1.31 (1.18 , 1.45)	1.40 (1.21 , 1.63)	1.43 (1.27 , 1.60)	0.99 (0.84 , 1.17)	1.17 (1.07 , 1.27)	1.15 (1.06 , 1.26)
Stage 3	1.18 (1.09 , 1.27)	1.19 (1.08 , 1.31)	1.39 (1.27 , 1.53)	1.83 (1.68 , 1.99)	1.82 (1.59 , 2.07)	1.72 (1.57 , 1.89)	1.42 (1.26 , 1.60)	1.42 (1.33 , 1.52)	1.37 (1.27 , 1.47)
Stage 4	1.24 (1.11 , 1.39)	1.14 (0.99 , 1.32)	1.39 (1.21 , 1.59)	2.09 (1.86 , 2.35)	1.82 (1.51 , 2.18)	1.94 (1.71 , 2.21)	1.85 (1.59 , 2.16)	1.44 (1.31 , 1.59)	1.47 (1.33 , 1.63)
Stage Missing	1.09 (1.01 , 1.18)	1.02 (0.92 , 1.12)	1.12 (1.02 , 1.24)	1.38 (1.26 , 1.52)	1.40 (1.22 , 1.61)	1.43 (1.29 , 1.57)	1.25 (1.11 , 1.42)	1.18 (1.10 , 1.27)	1.12 (1.04 , 1.20)
**Diagnosis (reference endometrium/uterus)**									
Vulva/Vagina	1.46 (1.31 , 1.64)	1.54 (1.34 , 1.78)	1.60 (1.40 , 1.82)	1.69 (1.49 , 1.93)	1.69 (1.40 , 2.05)	2.53 (2.25 , 2.83)	1.10 (0.90 , 1.33)	1.49 (1.35 , 1.65)	1.55 (1.40 , 1.71)
Cervix	1.32 (1.22 , 1.44)	1.34 (1.19 , 1.49)	1.77 (1.59 , 1.96)	2.03 (1.85 , 2.23)	2.29 (2.00 , 2.62)	1.80 (1.63 , 1.99)	1.23 (1.05 , 1.43)	1.61 (1.49 , 1.73)	1.39 (1.28 , 1.51)
Ovary/Fallopian Tube	1.00 (0.94 , 1.08)	1.09 (1.00 , 1.20)	1.14 (1.05 , 1.24)	1.40 (1.29 , 1.51)	1.33 (1.18 , 1.50)	1.08 (0.99 , 1.17)	1.25 (1.13 , 1.38)	1.16 (1.10 , 1.24)	1.11 (1.04 , 1.18)
**Material Resources Quintile (reference 5)**									
1	0.82 (0.75 , 0.89)	0.62 (0.56 , 0.70)	0.62 (0.56 , 0.69)	0.68 (0.62 , 0.75)	0.72 (0.62 , 0.83)	0.55 (0.50 , 0.61)	0.61 (0.53 , 0.70)	0.70 (0.65 , 0.75)	0.75 (0.70 , 0.82)
2	0.85 (0.78 , 0.93)	0.71 (0.64 , 0.79)	0.72 (0.65 , 0.80)	0.74 (0.67 , 0.81)	0.77 (0.67 , 0.89)	0.68 (0.61 , 0.75)	0.68 (0.60 , 0.78)	0.79 (0.73 , 0.85)	0.82 (0.76 , 0.88)
3	0.88 (0.81 , 0.96)	0.76 (0.68 , 0.84)	0.73 (0.66 , 0.81)	0.81 (0.74 , 0.89)	0.78 (0.68 , 0.89)	0.73 (0.66 , 0.81)	0.81 (0.71 , 0.92)	0.81 (0.76 , 0.88)	0.83 (0.77 , 0.90)

4	0.86 (0.79 , 0.94)	0.78 (0.70 , 0.87)	0.86 (0.78 , 0.96)	0.84 (0.76 , 0.92)	0.76 (0.66 , 0.88)	0.82 (0.74 , 0.90)	0.85 (0.75 , 0.97)	0.87 (0.81 , 0.94)	0.87 (0.80 , 0.94)
Immigrant (reference no)	0.90 (0.83 , 0.98)	1.29 (1.17 , 1.42)	0.95 (0.86 , 1.05)	1.13 (1.04 , 1.24)	1.13 (1.00 , 1.29)	1.33 (1.21 , 1.45)	1.27 (1.13 , 1.43)	1.01 (0.94 , 1.08)	1.24 (1.16 , 1.33)
Diagnosis year (per 1 year increase)	0.99 (0.99 , 1.00)	1.00 (0.99 , 1.01)	0.99 (0.99 , 1.00)	0.94 (0.93 , 0.95)	0.97 (0.96 , 0.99)	0.99 (0.98 , 1.00)	0.96 (0.95 , 0.97)	0.98 (0.98 , 0.99)	0.99 (0.98 , 1.00)
Months from diagnosis (per 6-month increase)	0.66 (0.64 , 0.68)	0.85 (0.82 , 0.88)	0.84 (0.82 , 0.87)	0.70 (0.67 , 0.72)	0.71 (0.67 , 0.75)	0.95 (0.92 , 0.98)	0.89 (0.85 , 0.93)	0.81 (0.79 , 0.83)	0.81 (0.79 , 0.83)
Level of comorbidity (reference 0-4)									
5-9	1.23 (1.08 , 1.39)	1.22 (1.03 , 1.44)	1.26 (1.08 , 1.48)	1.20 (1.05 , 1.37)	1.18 (0.96 , 1.44)	1.22 (1.04 , 1.43)	1.06 (0.87 , 1.30)	1.35 (1.21 , 1.51)	1.16 (1.04 , 1.30)
10-14	1.71 (1.52 , 1.93)	1.88 (1.60 , 2.21)	1.81 (1.55 , 2.11)	1.46 (1.28 , 1.67)	1.69 (1.39 , 2.06)	1.77 (1.52 , 2.07)	1.48 (1.22 , 1.80)	1.96 (1.75 , 2.18)	1.54 (1.38 , 1.73)
15-19	2.76 (2.42 , 3.14)	3.25 (2.74 , 3.85)	3.01 (2.56 , 3.55)	1.97 (1.70 , 2.27)	2.56 (2.06 , 3.17)	2.92 (2.48 , 3.43)	2.19 (1.78 , 2.70)	3.09 (2.75 , 3.48)	2.21 (1.96 , 2.50)
≥20	3.80 (3.00 , 4.81)	4.51 (3.38 , 6.01)	5.02 (3.84 , 6.57)	2.42 (1.85 , 3.16)	4.29 (2.94 , 6.25)	4.57 (3.46 , 6.05)	4.28 (3.09 , 5.93)	4.97 (4.05 , 6.10)	3.20 (2.57 , 3.98)

Table S6B. Multivariable logistic regression for odds of ESAS score >=4 (moderate-to-severe) [aOR (95% CI)].

	Anxiety	Depress-ion	Drowsi-ness	Appetite	Nausea	Pain	Dyspnea	Tired-ness	Well-being
**Age at diagnosis (per 10-year increase)**	0.85 (0.83 , 0.86)	0.87 (0.85 , 0.89)	0.94 (0.92 , 0.96)	0.99 (0.97 , 1.01)	0.84 (0.82 , 0.86)	0.94 (0.92 , 0.96)	1.11 (1.08 , 1.13)	0.95 (0.93 , 0.96)	0.92 (0.91 , 0.94)
**Disease stage (TNM) (reference 1)**									
Stage 2	1.05 (0.98 , 1.12)	1.12 (1.04 , 1.21)	1.20 (1.12 , 1.29)	1.29 (1.20 , 1.38)	1.57 (1.43 , 1.73)	1.32 (1.22 , 1.42)	1.07 (0.98 , 1.18)	1.19 (1.12 , 1.26)	1.21 (1.14 , 1.29)
Stage 3	1.23 (1.17 , 1.31)	1.33 (1.25 , 1.42)	1.45 (1.36 , 1.54)	1.70 (1.60 , 1.80)	1.88 (1.73 , 2.04)	1.62 (1.53 , 1.73)	1.41 (1.31 , 1.52)	1.46 (1.39 , 1.54)	1.47 (1.39 , 1.55)
Stage 4	1.34 (1.23 , 1.47)	1.43 (1.30 , 1.58)	1.53 (1.39 , 1.67)	1.97 (1.81 , 2.15)	1.96 (1.74 , 2.20)	1.86 (1.70 , 2.04)	1.77 (1.59 , 1.97)	1.52 (1.41 , 1.65)	1.67 (1.54 , 1.81)
Stage Missing	1.13 (1.07 , 1.19)	1.13 (1.06 , 1.21)	1.13 (1.06 , 1.20)	1.30 (1.22 , 1.38)	1.37 (1.25 , 1.49)	1.27 (1.19 , 1.35)	1.17 (1.08 , 1.26)	1.15 (1.09 , 1.21)	1.17 (1.11 , 1.23)
**Diagnosis (reference endometrium/ uterus)**									
Vulva/Vagina	1.25 (1.15 , 1.36)	1.33 (1.21 , 1.47)	1.50 (1.37 , 1.64)	1.51 (1.38 , 1.65)	1.60 (1.41 , 1.81)	2.19 (2.01 , 2.39)	1.18 (1.05 , 1.33)	1.39 (1.29 , 1.51)	1.44 (1.33 , 1.55)
Cervix	1.22 (1.15 , 1.30)	1.27 (1.18 , 1.37)	1.60 (1.50 , 1.72)	1.85 (1.74 , 1.98)	1.99 (1.83 , 2.17)	1.52 (1.42 , 1.63)	1.26 (1.15 , 1.37)	1.49 (1.40 , 1.58)	1.36 (1.29 , 1.45)
Ovary/Fallopian Tube	1.02 (0.97 , 1.08)	1.05 (0.99 , 1.12)	1.11 (1.05 , 1.18)	1.23 (1.17 , 1.30)	1.24 (1.15 , 1.33)	1.17 (1.11 , 1.24)	1.24 (1.16 , 1.32)	1.14 (1.09 , 1.19)	1.14 (1.09 , 1.19)
**Material Resources Quintile (reference 5)**									
1	0.85 (0.80 , 0.90)	0.69 (0.64 , 0.74)	0.70 (0.66 , 0.75)	0.73 (0.69 , 0.78)	0.73 (0.66 , 0.80)	0.61 (0.57 , 0.66)	0.64 (0.59 , 0.70)	0.75 (0.70 , 0.79)	0.80 (0.76 , 0.85)
2	0.88 (0.83 , 0.94)	0.75 (0.70 , 0.81)	0.76 (0.71 , 0.82)	0.79 (0.74 , 0.85)	0.78 (0.71 , 0.85)	0.71 (0.66 , 0.76)	0.68 (0.63 , 0.74)	0.79 (0.75 , 0.84)	0.82 (0.78 , 0.87)
3	0.89 (0.83 , 0.94)	0.79 (0.74 , 0.85)	0.81 (0.76 , 0.87)	0.83 (0.78 , 0.89)	0.79 (0.72 , 0.86)	0.78 (0.73 , 0.83)	0.82 (0.75 , 0.89)	0.85 (0.80 , 0.90)	0.84 (0.79 , 0.89)
4	0.86 (0.81 , 0.92)	0.78 (0.72 , 0.84)	0.88 (0.82 , 0.94)	0.85 (0.80 , 0.91)	0.81 (0.74 , 0.89)	0.82 (0.76 , 0.87)	0.81 (0.74 , 0.88)	0.89 (0.84 , 0.94)	0.85 (0.81 , 0.91)

Immigrant (reference no)	0.84 (0.79 , 0.89)	1.24 (1.16 , 1.32)	0.93 (0.88 , 0.99)	1.03 (0.97 , 1.10)	1.11 (1.03 , 1.21)	1.40 (1.31 , 1.49)	1.21 (1.13 , 1.31)	0.97 (0.92 , 1.03)	1.13 (1.07 , 1.19)
Diagnosis year (per 1 year increase)	0.99 (0.99 , 1.00)	0.99 (0.99 , 1.00)	1.00 (0.99 , 1.00)	0.93 (0.92 , 0.93)	0.99 (0.98 , 0.99)	0.99 (0.99 , 1.00)	0.97 (0.97 , 0.98)	0.98 (0.98 , 0.99)	0.99 (0.98 , 1.00)
Months from diagnosis (per 6-month increase)	0.70 (0.69 , 0.72)	0.86 (0.85 , 0.88)	0.84 (0.82 , 0.86)	0.70 (0.68 , 0.71)	0.76 (0.74 , 0.79)	0.90 (0.88 , 0.92)	0.92 (0.90 , 0.94)	0.79 (0.78 , 0.80)	0.78 (0.76 , 0.79)
Level of comorbidity (reference 0-4)									
5-9	1.21 (1.11 , 1.31)	1.30 (1.17 , 1.45)	1.20 (1.09 , 1.32)	1.22 (1.12 , 1.34)	1.22 (1.07 , 1.38)	1.21 (1.10 , 1.34)	1.25 (1.10 , 1.42)	1.30 (1.20 , 1.40)	1.21 (1.12 , 1.31)
10-14	1.67 (1.54 , 1.82)	1.93 (1.74 , 2.14)	1.72 (1.56 , 1.89)	1.55 (1.42 , 1.69)	1.61 (1.42 , 1.82)	1.76 (1.59 , 1.94)	1.77 (1.57 , 2.01)	1.87 (1.74 , 2.02)	1.74 (1.61 , 1.88)
15-19	2.47 (2.25 , 2.70)	3.15 (2.82 , 3.52)	2.62 (2.36 , 2.90)	2.09 (1.90 , 2.31)	2.41 (2.10 , 2.76)	2.84 (2.56 , 3.16)	2.62 (2.29 , 2.99)	2.88 (2.65 , 3.14)	2.60 (2.39 , 2.83)
≥20	3.35 (2.78 , 4.04)	4.68 (3.82 , 5.74)	4.18 (3.43 , 5.10)	2.89 (2.39 , 3.51)	4.09 (3.15 , 5.30)	4.84 (3.98 , 5.88)	4.27 (3.38 , 5.40)	5.11 (4.20 , 6.20)	4.10 (3.43 , 4.90)
